# Enhancing efficiency and stability in perovskite solar cells: innovations in self-assembled monolayers

**DOI:** 10.3389/fchem.2024.1519166

**Published:** 2025-01-06

**Authors:** Jingshu Tian, Haichang Zhang

**Affiliations:** Key laboratory of Rubber-Plastic of Ministry of Education /Shandong Province (QUST), School of Polymer Science and Engineering, Qingdao University of Science and Technology, Qingdao, China

**Keywords:** perovskite solar cell (PSC), self-assemble layer, stability, power conversion efficiency (PCE), interface engineering

## Abstract

Perovskite solar cells (PVSCs) show remarkable potential due to their high-power conversion efficiencies and scalability. However, challenges related to stability and long-term performance remain significant. Self-assembled monolayers (SAMs) have emerged as a crucial solution, enhancing interfacial properties, facilitating hole extraction, and minimizing non-radiative recombination. This review examines recent advancements in SAMs for PVSCs, focusing on three key areas: anchoring groups and interface engineering, electronic structure modulation as well as band alignment, and stability optimization. We emphasize the role of anchoring groups in reducing defects and improving crystallinity, alongside the ability of SAMs to fine-tune energy levels for more effective hole extraction. Additionally, co-adsorbed SAM strategies was discussed which can enhance the durability of PVSCs against thermal and moisture degradation. Overall, SAMs present a promising avenue for addressing both efficiency and stability challenges in PVSCs, paving the way toward commercial viability. Future research should prioritize long-term environmental durability and the scaling up of SAM applications for industrial implementation.

## 1 Introduction

Perovskite solar cells (PVSCs) have gained widespread attention as one of the most promising candidates for next-generation photovoltaic technology, combining high power conversion efficiency (PCE) with the potential for cost-effective, scalable production (Dai et al., 2021; Aktas et al., 2020; Caprioglio et al., 2023). Significant progress has been made in improving the efficiency of PVSCs over the past decade, with certified PCEs now comparable to those of conventional silicon-based solar cells (Shen et al., 2023; Sun et al., 2022; Galvis et al., 2023). Despite these achievements, significant challenges remain, particularly regarding the stability of PVSCs and the need for more efficient charge extraction and transport at the interface between the perovskite layer and the charge transport layer (Artiom et al., 2018; Gao et al., 2024; Jiang et al., 2024a). The charge transport layer (CTL) plays a crucial role in perovskite solar cells. It is located between the perovskite layer and other functional layers and is primarily responsible for transporting charges, ensuring that charges can be efficiently extracted from the perovskite layer and transported to the electrodes (Li B et al., 2024; Liao et al., 2022a; Liu et al., 2015).

In addition, the charge selective layer (CSL) is a crucial component in PVSCs, which is located above or below the perovskite layer to optimize charge extraction and transport (Marchant and Williams, 2024; Phung et al., 2022; Sekimoto et al., 2023). (i) Efficient extraction of charge: the main task of the charge extraction layer is to efficiently extract the charges (electrons or holes) generated in the photogeneration process from the active layer (such as the perovskite layer) and transfer them to the electrode (Tang et al., 2024; Wang et al., 2020; Wu et al., 2022). (ii) Reduce recombination losses: If the electrons and holes generated in the active layer cannot be extracted in time, they may recombine in the layer, resulting in the loss of photogenerated current. The CSL reduces this recombination loss by providing an efficient transmission channel (Yao et al., 2022; Zhu et al., 2022; Zhang et al., 2023). (iii) Improve interface characteristics: The CSL can optimize the interface characteristics between the active layer and the electrode, reduce the interface resistance and potential barrier, and thus improve the performance of the overall device (Yeo et al., 2024; Wojciechowski et al., 2014; Li W et al., 2023). (iv) Block reverse charge: In some designs, the charge extraction layer can also play a role in blocking reverse charge, preventing unwanted charge flow and further improving the stability of the device (Liao et al., 2022b; Lin et al., 2024; Park et al., 2023). (v) Regulate the band structure: The material selection and thickness of the charge extraction layer can be used to regulate the band structure to ensure that electrons and holes can be smoothly transferred from the active layer to the electrode (Chang et al., 2021; Han and Zhang, 2024; Hung et al., 2023). (vi) Protect the active layer: The charge extraction layer can also play a role in protecting the active layer to prevent damage to the active layer by the external environment (such as oxygen, moisture, etc.) (Hou et al., 2018; Huang et al., 2024; Deng et al., 2022).

In inverted PVSCs, the use of ultrathin self-assembled monolayers (SAMs) that can effectively extract holes from perovskites to the anode has become increasingly popular as hole-selective layers (HSLs) (Cassella et al., 2022; Aydin et al., 2023; Dai et al., 2022). Unlike PTAA or other traditional organic hole-transporting materials, SAM (Self-Assembled Monolayer) materials are characterized by their extremely low consumption, simple manufacturing process, and the ability to fine-tune their chemical structures at the molecular level (Hsu et al., 2024; Li D et al., 2024; Valles-Pelarda et al., 2016). Consequently, SAM materials hold great promise as an economical, scalable, and stable Hole-Selective Layer (HSL) for inverted PVSCs. By optimizing the molecular structure of SAMs, researchers can significantly enhance interfacial energy levels, improve hole extraction, reduce non-radiative recombination, and ultimately boost the overall efficiency and stability of PVSCs (Liu L et al., 2023; Li Z et al., 2023; Jiang et al., 2022).

This review delves into the recent breakthroughs in the design and deployment of SAMs within PVSCs, with a concerted focus on three pivotal areas. Initially, we scrutinize the influence of anchoring groups and interface engineering, elucidating how diverse functional groups and molecular configurations can augment adhesion, mitigate interfacial defects, and bolster overall device efficacy. Subsequently, we investigate electronic structure modulation and band alignment, concentrating on strategic design approaches that refine energy level matching and enhance charge transport at interfaces. Lastly, we address the optimization of stability and durability, underscoring the engineering methodologies that ensure SAM materials maintain long-term operational resilience across a spectrum of environmental stressors, such as elevated temperatures, humidity, and extended light exposure. Through an extensive examination of these domains, this review aspires to furnish a detailed understanding of how SAMs can significantly contribute to surmounting the existing challenges faced by PVSCs, thereby facilitating their widespread adoption in the evolving landscape of sustainable energy.

### 1.1 Anchoring groups and interface engineering

Anchoring groups hold a pivotal role in the optimization of interfaces within perovskite solar cells, exerting a direct influence on charge extraction and significantly reducing recombination losses (Kim et al., 2024; Li B et al., 2024; Lin et al., 2017). The meticulous selection of an appropriate anchoring group is imperative, as it governs the molecular interactions between self-assembled monolayers and the perovskite layer, thereby dictating the overall performance characteristics of the device (Jiang et al., 2023; Ernestas et al., 2024). This section delves into the ramifications of employing various anchoring groups, elucidating how their strategic choice can result in enhanced adhesion, effective defect passivation, and precise energy level alignment. These factors collectively contribute to elevated power conversion efficiencies and improved device stability, underscoring the critical importance of anchoring group selection in the advancement of PVSCs technology (Jiang et al., 2024b; He et al., 2023).

**FIGURE 1:**
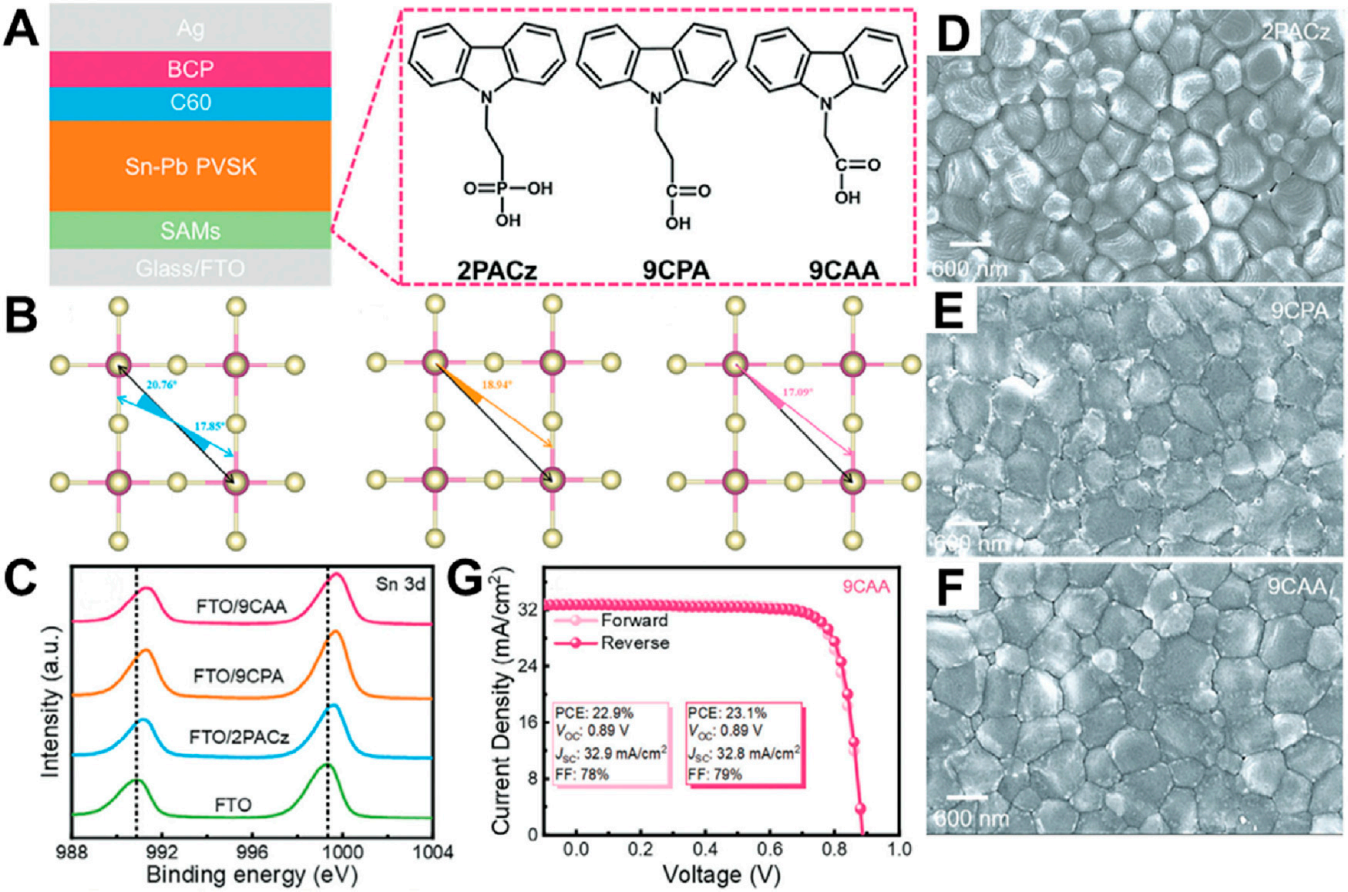
**(A)** The device structure of FTO/SAM/Sn–Pb perovskite (Cs_0.1_FA_0.6_MA_0.3_Pb_0.5_Sn_0.5_I_3_)/C_60_/BCP/Ag and molecular structure of 2PACz, 9CPA, and 9CAA. **(B)** The deviation angles of 2PACz/I atoms, 9CPA/I atom, and 9CAA/I atom. **(C)** XPS spectra of Sn 3days of FTO, FTO/2PACz, FTO/9CPA, and FTO/9CAA. Top SEM images of **(D)** 2PACz-, **(E)** 9CPA-, and **(F)** 9CAA-based perovskite films. **(G)** *J*–*V* curves of 9CAA-based devices (Zhang et al., 2024).

Three distinct SAM molecules—2PACz, 9CPA, and 9CAA—were investigated as hole-selective layers (HSLs) in Sn-Pb perovskite solar cells (PVSCs) (Zhang et al., 2024). As illustrated in Figure 1A, these SAMs exhibit variations in their anchoring groups and molecular architectures. Specifically, 2PACz features a phosphonic acid group, whereas 9CPA and 9CAA contain carboxylate groups. These differences in anchoring chemistry substantially influence the manner in which these molecules adhere to the substrate and engage with the perovskite layer. The orientation of these SAMs is totally different which might be caused by the various anchoring groups induced diverse molecular self-assemble. In addition, the deviation angles of SAMs/I atoms are totally different. There is only a single angle between the carboxylate groups-based SAMs/I atoms (18.94° for 9CPA, Figure 1B middle; 17.09° for 9CAA; Figure 1B right), but two deviation angles for the phosphonic acid-based SAM/I atoms (17.85° and 20.76° for 2PACz, Figure 1B left). 9CAA exhibits the most advantageous alignment due to its smallest SAM/I atom angles. This enhanced orientation fosters improved crystallinity within the perovskite layer, as evidenced by the observations in Figures 1D–F. Consequently, this leads to a reduction in defects and diminished recombination losses. The superior molecular arrangement and decreased defect density directly contribute to more effective charge extraction, thereby elevating overall device performance.

Furthermore, the X-ray photoelectron spectroscopy (XPS) results presented in Figure 1C underscore the superior passivation of the Sn 3d orbital in the 9CAA-modified samples, indicative of stronger binding and more efficacious defect passivation at the interface. This enhanced passivation contributes to improved hole extraction efficiency, as evidenced by the overall device performance metrics. The 9CAA-modified solar cells achieved a record-high power conversion efficiency (PCE) of 23.1%, accompanied by an open-circuit voltage (*V*_OC_) of 0.89 V and a short-circuit current density (*J*_SC_) of 32.8 mA/cm^2^, as depicted in Figure 1G. These findings highlight the significant impact of optimized molecular arrangement and anchoring chemistry on interface quality, ultimately leading to superior device performance. Despite these promising results, additional research is necessary to evaluate the long-term stability of these SAM-modified devices under real-world operational conditions, including temperature fluctuations, moisture exposure, and extended light exposure. Future investigations should prioritize the durability of SAM-based interfaces and explore strategies to bolster their environmental robustness.

**FIGURE 2:**
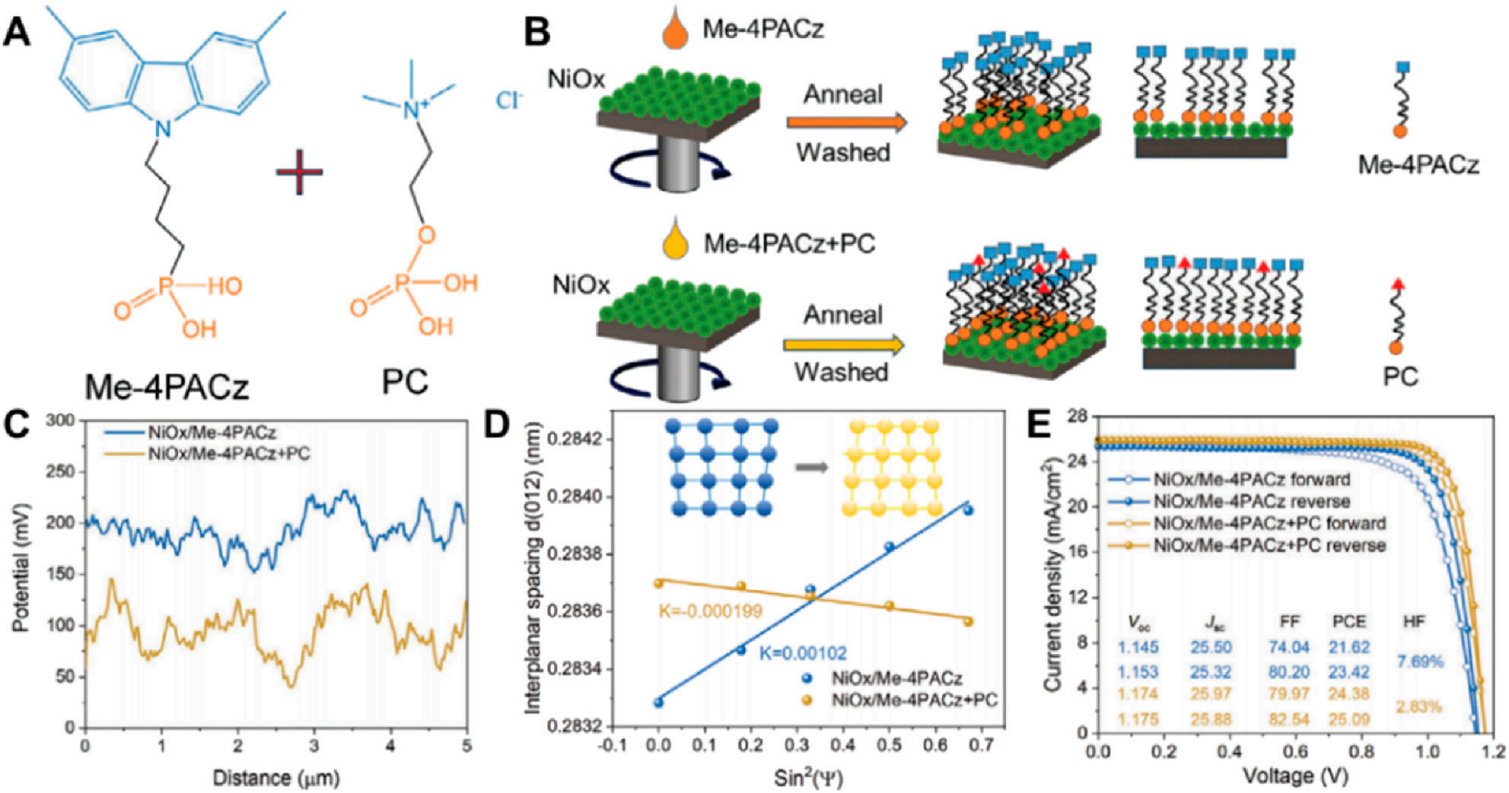
**(A)** Molecular structures of Me-4PACz and PC. **(B)** Schematic illustration of SAM (Me-4PACz) and Co-SAM (Me-4PACz + PC) of modified NiOx. **(C)** CPD changes of NiOx/Me-4PACz and NiOx/Me-4PACz + PC films. **(D)** D-spacing values obtained from (012) plane as a function of incidence angle (interior illustration: schematic of the transformation from tensile stress to compressive stress) **(E)** The forward and reverse scanning performance of NiOx-deposited Me-4PACz and Me-4PACz + PC devices (Cao et al., 2024).

Expanding upon the effective interface passivation observed with 9CAA, further progress can be achieved by addressing the surface defects present in metal oxide-based hole transport layers (HTLs). The introduction of the co-self-assembled monolayer (Co-SAM) strategy, which combines Me-4PACz and phosphorylcholine chloride (PC) to modify NiOx HTLs, has been shown to enhance both interface coverage and defect passivation (Cao et al., 2024). As illustrated in Figure 2A, Feng Yan’s research team observed that the co-assembly of Me-4PACz and PC on the NiOx surface yields a good complete and uniform monolayer. While Me-4PACz alone contributes to interface improvement, it does not achieve complete surface passivation. The incorporation of PC serves to fill organic cation and halide vacancies, thereby facilitating superior interface passivation, as depicted in the surface modification schematics in Figure 2B. The surface contact potential difference (CPD) was measured. As depicted in Figure 2C , the Co-SAM-modified NiOx (NiOx/Me-4PACz + PC) exhibits a large amplitude of fluctuation and low potential distribution in comparison to NiOx/Me-4PACz one. This observation signifies a substantial reduction in leakage current and an enhancement in device stability. Additional confirmation of these improvements is provided by the X-ray diffraction data presented in Figure 2D, which reveals a smaller interplanar spacing in the Co-SAM-modified sample. This finding suggests improved molecular packing, which serves to minimize non-radiative recombination and enhance charge transport efficiency. The device performance metrics, as illustrated in Figure 2E, reflect these advancements. The Co-SAM-modified NiOx devices achieved a power conversion efficiency of 25.09%, outperforming the 23.42% efficiency of devices modified solely with Me-4PACz. Enhancements in fill factor (FF) and open-circuit voltage, coupled with reduced hysteresis, further underscore the benefits of employing this co-assembly strategy. While the Co-SAM approach markedly improves short-term performance, additional research is warranted to assess the long-term environmental stability of these devices, particularly under conditions of elevated temperature and moisture exposure.

**FIGURE 3:**
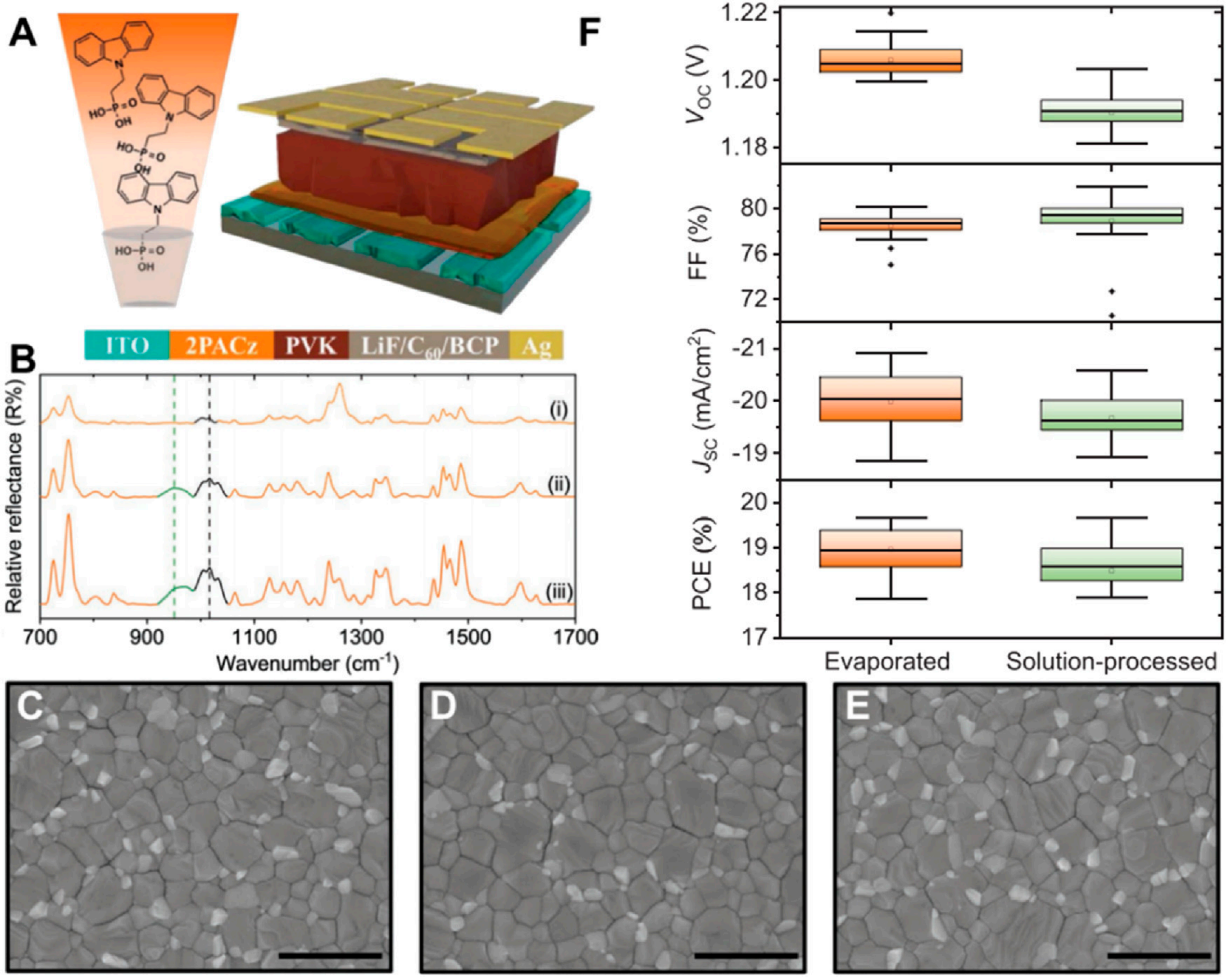
**(A)** Schematic diagram of the device stack employing triple-cation perovskite (PVSK) composition (Cs_0.17_FA_0.83_PbI_2.75_Br_0.25_) and evaporated 2PACz hole transport layer (HTL). Also shown is a rendering of evaporation process. **(B)** Reflection–absorption infrared spectra of evaporated 2PACz thin films with different thickness onto glass/ITO substrates. The green dashed line represents the peak position of P-OH vibration band of the bulk 2PACz. **(C–E)** Top view scanning electron microscope images of perovskite thin films deposited over **(C)** solution-processed 2PACz layer, **(D)** ≈6 nm evaporated 2PACz layer, and **(E)** ≈200 nm evaporated and washed 2PACz layer, respectively. The scale bar is 1 µm. **(F)** Statistical distribution of the FF, J_SC_, V_OC_, and PCE of perovskite solar cells employing evaporated (≈6 nm) and solution-processed 2PACz HTLs (Farag et al., 2023).

Building upon preceding research that has utilized SAMs to refine perovskite solar cell interfaces, Ahmed Farag and his team compare studied the SAMs preparation techniques between the solution method and the vacuum-based evaporation method (Farag et al., 2023). 2PACz (Figure 3A) was applied using vacuum evaporation techniques, with its performance compared to that of solution-processed 2PACz. Infrared (IR) spectra depicted in Figure 3B affirm that both the evaporation and solution processing methods maintain comparable chemical structures. Furthermore, SEM images presented in Figures 3C–E reveal that both methodologies yield smooth and compact perovskite layers with minimal defects—a characteristic imperative for enhancing device performance. As illustrated in Figure 3F, underscores the benefits of employing vacuum evaporation technique. This method exhibited a marginally superior power conversion efficiency of 19.6%, outperforming the solution-processed PCE of 18.5%. Additionally, evaporated 2PACz demonstrated enhancements in both open-circuit voltage and fill factor, thereby highlighting its potential for facilitating nearly lossless interfaces and mitigating recombination losses. Nevertheless, while the evaporated approach improves interface quality and device efficiency, it necessitates further scrutiny regarding long-term stability and scalability for industrial applications.

### 1.2 Electronic structure modulation and band alignment

**FIGURE 4:**
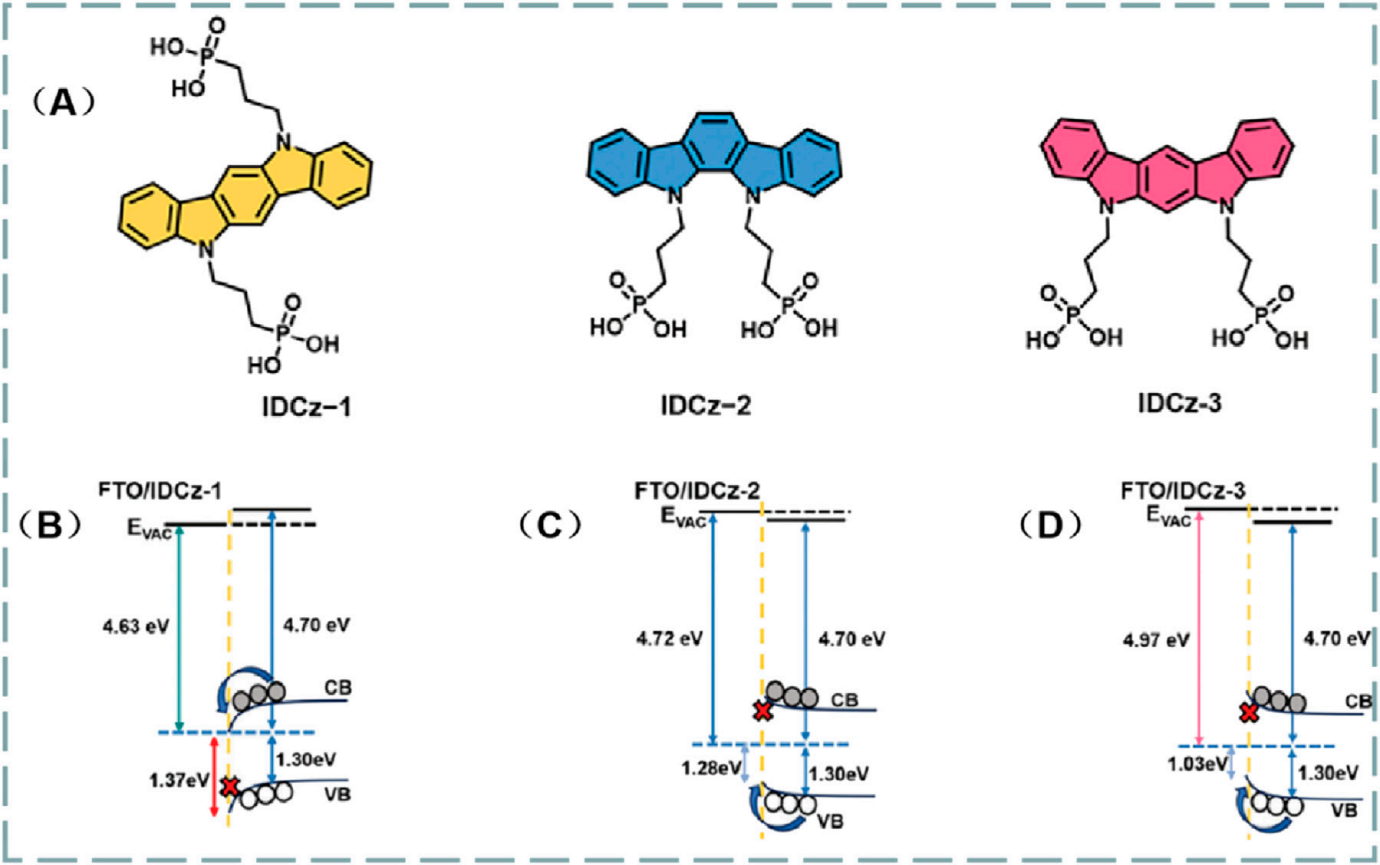
**(A)**, Chemical structures of the three molecules IDCz-1, -2 and -3. Energy-level diagrams for FTO/IDCz-1/perovskite **(B)**, FTO/IDCz-2/perovskite **(C)**, and FTO/IDCz-3/perovskite **(D)** (Wu et al., 2024).

In PVSCs, the precise alignment of energy levels between the perovskite absorber and the charge transport layers emerges as a pivotal determinant of charge transfer efficiency and, consequently, overall device performance (Feleki et al., 2022). Attaining this optimal energy alignment is crucial as it minimizes energy losses, streamlines efficient charge extraction, and curtails non-radiative recombination. Suitable energy levels might result the Ohmic contact formation, which is advantage for the chare extraction and transporting. Self-assembled monolayers (SAMs) present a robust framework for modulating the electronic structure at these interfaces, enabling the fine-tuning of work functions and dipole moments to foster more conducive conditions for charge transport (Zhong et al., 2024; Wang J et al., 2024; Zuo et al., 2017). Wu et al. designed three bisphosphonate-anchored indolocarbazole (IDCz)-based SAMs, namely IDCZ-1, -2, and -3, which are isomers (Figure 4A). By adjusting the positions of the nitrogen atoms in the IDCz core, they modified the molecular dipole moments and π-π interactions (Wu et al., 2024). Molecular design plays a crucial role in tuning the work function and energy level alignment at the interface, as evidenced in Figure 4B. The WFs of the bare FTO were −4.58 eV, significantly higher than those modified by the designed SAMs (Figures 4B–D; −4.63 for IDCz-1, -4.72 for IDCz-2, and -4.97 eV for IDCz-3). These differences in WFs can be attributed to the varying dipole moments of the materials (0.98 D for IDCz-1, 1.41 D for IDCz-2, and 4.27 D for IDCz-3). Lower WFs are more advantageous for hole extraction while blocking electrons, whereas higher WFs favor electron extraction. Consequently, the PCEs of the devices based on these three materials were 20.97% (IDCz-1), 23.11% (IDCz-2), and 25.15% (IDCz-3), respectively. These devices were configured as FTO/SAM/Cs0.05FA0.85MA0.1PbI3/piperazinium iodide (PI)/C60/bathocuproine (BCP)/Ag. Compared to the devices based on IDCz-1 and -2, the IDCz-3-based device exhibited significantly improved performance, likely due to enhanced energy level matching and reduced recombination losses. Further insights were provided by photovoltage decay measurements, which revealed that IDCz-3 exhibited the longest carrier lifetime (*τ* = 1,302 ns). This indicates reduced trap-assisted recombination and more efficient charge transport compared to the other two SAM variants (728 ns for IDCz-1 and 1,171 ns for IDCz-2, respectively). Although the molecular modifications significantly enhanced performance, additional studies are necessary to investigate the long-term stability of these materials under operational conditions, such as exposure to moisture and light stress.

**FIGURE 5:**
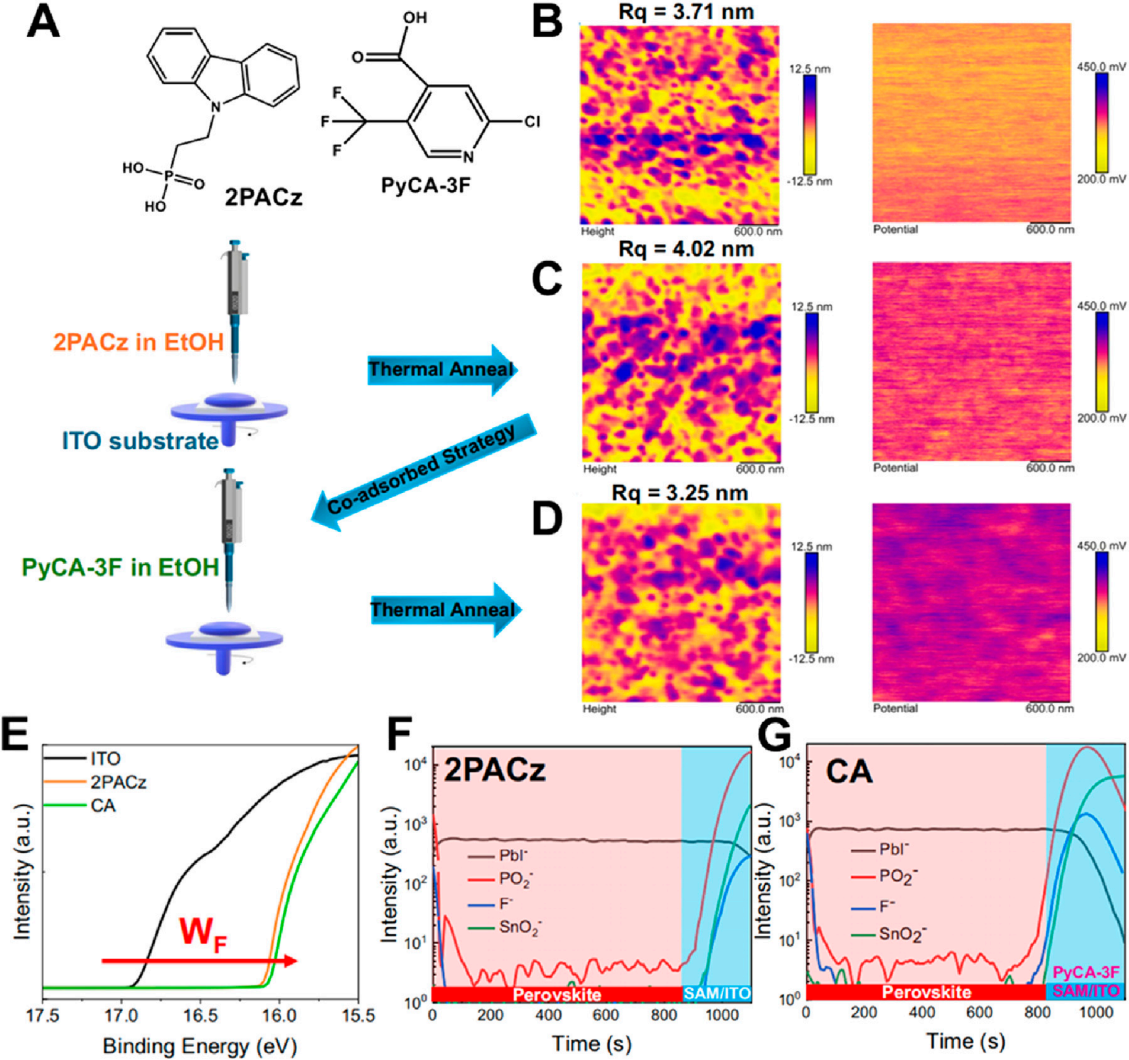
**(A)** Chemical structures of 2PACz and PyCA-3F, and schematic diagram of HTL/SAM fabrication. **(B)** The AFM height images and KPFM images of ITO substrates. **(C)** The AFM height images and KPFM images of 2PACz substrates. **(D)** The AFM height images and KPFM images of CA substrates. **(E)** UPS spectra of ITO, 2PACz, and CA substrates. **(F)** TOF-SIMS images of the buried interface of 2PACz-based perovskite film. **(G)** TOF-SIMS images of the top surface of CA-based perovskite film (Li et al., 2024).

Following the encouraging outcomes associated with evaporated self-assembled monolayers, additional optimization can be attained by amalgamating distinct SAM molecules to bolster interface passivation. Chun Cheng’s team delved into a co-adsorbed SAM tactic that integrates both 2PACz and PyCA-3F, as delineated in Figure 5A (Li D et al., 2024). The co-adsorption of these two molecules elevates the interface between the ITO substrate and the perovskite layer. As evidenced in Figures 5B–D, atomic force microscopy illustrates that the co-adsorbed SAM yields a smoother surface (R_q_ = 3.25 nm) in contrast to single-layer SAMs, thereby diminishing surface roughness and enhancing interface uniformity. Such a smoother surface is pivotal for minimizing defect states and augmenting charge transport across the interface. Work function measurements depicted in Figure 5E further underscore the efficacy of the co-adsorbed approach. The amalgamation of 2PACz and PyCA-3F modulates the energy levels more proficiently than 2PACz in isolation, optimizing the energy alignment at the interface. This leads to a reduction in recombination losses and fosters improved charge extraction, ultimately boosting overall device performance. Moreover, time-of-flight secondary ion mass spectrometry results presented in Figures 5F, G reveal deeper and more consistent penetration of PyCA-3F into the interface, thereby further refining passivation and curtailing interface defects. The refined energy alignment and enhanced charge transport attributes contribute to increased power conversion efficiency and mitigated recombination. Although the co-adsorbed SAM strategy exhibits distinct advantages in terms of defect passivation and device performance enhancement, additional research is warranted to evaluate its long-term stability under operational conditions.

**FIGURE 6:**
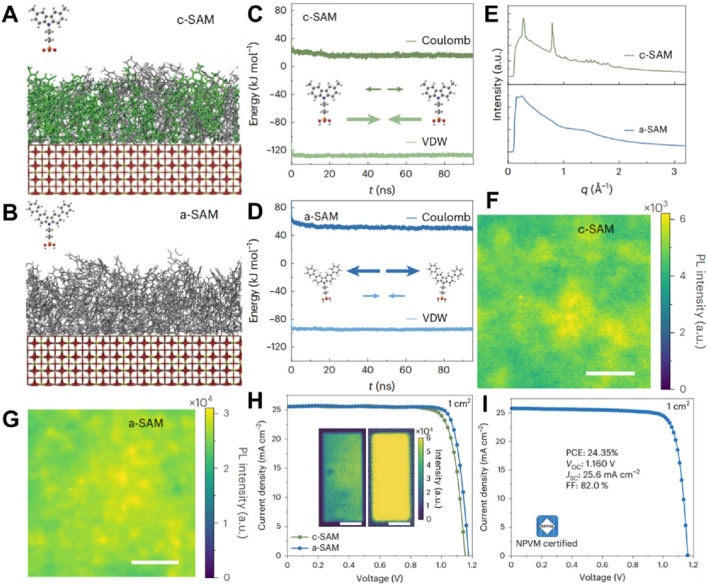
**(A)** MD simulation results of a side view of c-SAM absorbed on TCOs (50 ns, 6 nm × 6 nm). **(B)** MD simulation results of a side view of a-SAM absorbed on TCOs (50 ns). **(C)** MD-simulated Coulomb and van der Waals interaction energies of c-SAM. **(D)** MD-simulated Coulomb and van der Waals interaction energies of a-SAM. **(E)** Radial GIWAXS profiles integrated over all azimuthal angles (pseudo-X-ray diffraction profiles) for c-SAM and a-SAM. **(F, G)** PL intensity maps of the encapsulated perovskite film on c-SAM and a-SAM. **(H)** *J*–*V* scans of 1-cm2 c-SAM and a-SAM PVSCs. Inset: electroluminescence mapping of c-SAM (left) and a-SAM (right) PVSCs. Scale bars, 5 mm. **(I)** *J–V* scan measurement of the 1-cm2 device recorded by NPVM (Wang X et al., 2024).

Expanding upon the achievements related to co-adsorbed self-assembled monolayers, Yi Hou and his colleagues investigate how manipulating the molecular arrangement and phase homogeneity of SAMs can further boost the performance of perovskite solar cells (Wang X et al., 2024). A comparison between crystalline (c-SAM) and amorphous (a-SAM) monolayers is depicted in Figures 6A, B, where the orderly structure of c-SAM stands in contrast to the disordered configuration of a-SAM. Molecular dynamics simulations illustrated in Figures 6C, D emphasize the disparities in interaction energies, with c-SAM exhibiting stronger van der Waals (VDW) forces and Coulombic interactions in comparison to a-SAM. This augmented interaction strength inherent in c-SAM culminates in a more stable interface, which is crucial for effective charge extraction and the mitigation of recombination losses. The ramifications of molecular ordering are further exemplified in the photoluminescence intensity maps portrayed in Figures 6F, G. The more consistent distribution of PL intensity within c-SAM signifies superior phase homogeneity, which in turn correlates with fewer trap states and enhanced charge transport across the interface. Device performance assessments depicted in Figures 6H, I reveal that devices modified with c-SAM achieved a certified power conversion efficiency of 24.35%, coupled with improved open-circuit voltage and short-circuit current density in comparison to a-SAM. The enhanced performance observed in c-SAM is ascribed to its ordered molecular structure, which fosters better energy alignment and diminishes recombination losses. Despite c-SAM’s clear advantages in terms of efficiency and phase stability, subsequent research is necessary to appraise its long-term operational stability when subjected to environmental stressors, such as exposure to moisture and fluctuations in temperature.

### 1.3 Stability and durability optimization

**FIGURE 7:**
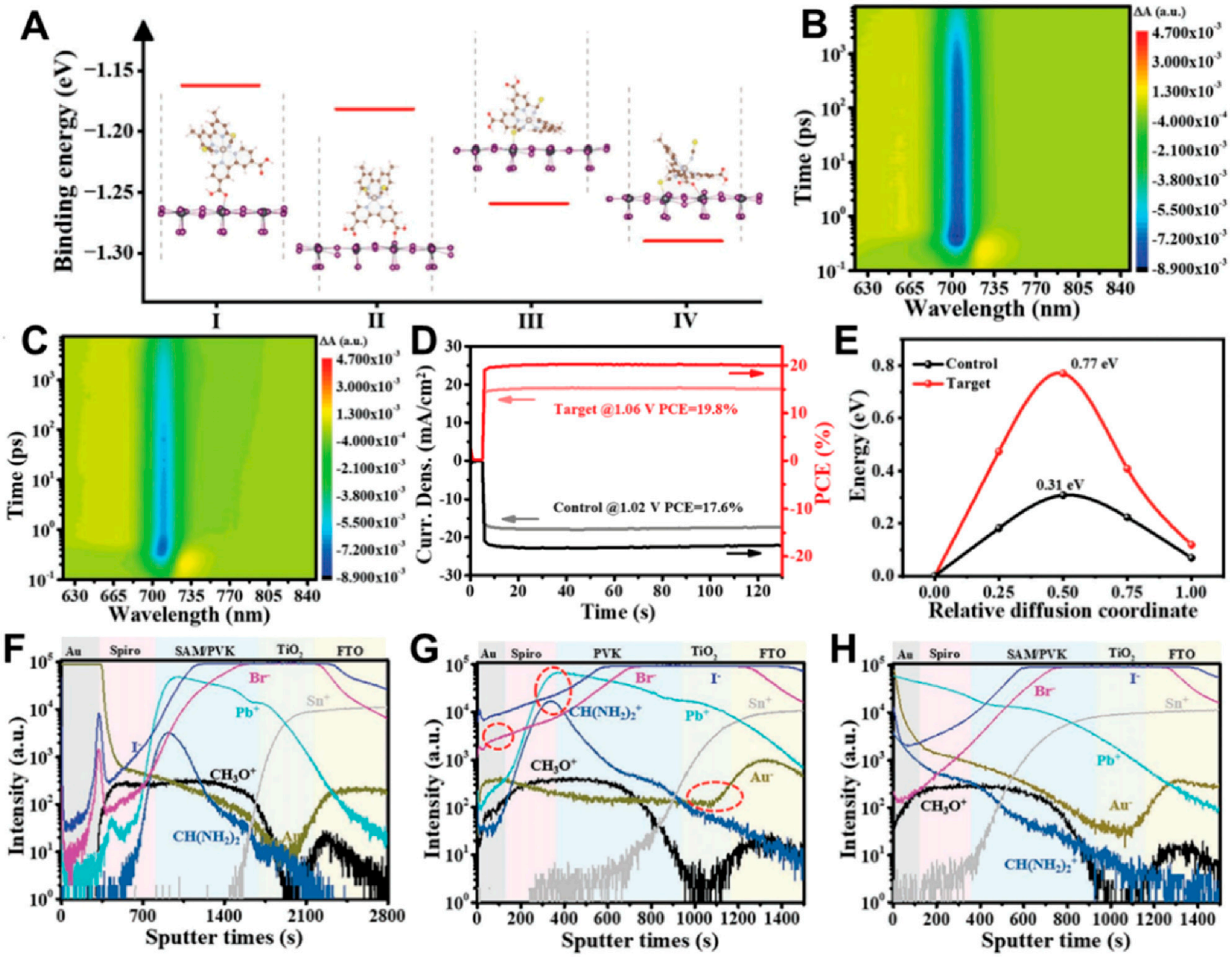
**(A)** Binding energies of various energetically favorable conformations. **(B, C)** Pseudocolor TA plots. **(D)** Steady-state power output of control and target devices. **(E)** The Ea of the halide ion migration on the perovskite surface with and without the SAM. ToF-SIMS depth profiles of **(F)** fresh device, **(G)** control, and **(H)** target devices after aging at 60% RH, 25°C for 600 h (Liu L et al., 2023).

Stability continues to be one of the most formidable obstacles to the commercialization of perovskite solar cells (PVSCs). Although significant strides have been made in attaining high power conversion efficiencies, the long-term operational stability of PVSCs, particularly under real-world environmental conditions such as heat, moisture, and extended light exposure, remains a substantial challenge (Zhu et al., 2020; Xu et al., 2024; Wolff et al., 2020). The employment of self-assembled monolayers (SAMs) presents a promising approach to bolster the durability of PVSCs by offering enhanced surface passivation and safeguarding the delicate perovskite layer from degradation (Sun et al., 2024; Tong et al., 2024; Seo et al., 2020). Building upon prior discussions regarding SAM-modified interfaces, Shengzhong Liu’s team delves into how SAMs can be utilized to meticulously tune energy alignment between the perovskite and charge transport layers (Liu M et al., 2023). Figure 7A illustrates how SAM-modified interfaces modulate the binding energy at the perovskite layer, thereby influencing charge extraction efficiency. The time-resolved spectra depicted in Figures 7B,C demonstrate faster and more efficient charge extraction in SAM-modified devices, which has a direct impact on device performance. Figure 7D juxtaposes the current density-voltage characteristics of a target device (featuring optimized SAM layers) with those of a control device. The target device achieves a power conversion efficiency (PCE) of 19.8% with an open-circuit voltage (*V*_OC_) of 1.06 V, surpassing the control device, which has a PCE of 17.6% and a *V*_OC_ of 1.02 V.

The energy barrier, as illustrated in Figure 7E, is significantly diminished in the SAM-modified device, facilitating improved charge transport across the interface. This optimization of energy alignment results in enhanced device efficiency and stability. Further chemical composition analysis depicted in Figures 7F–H reveals deeper penetration of key elements (such as Pb and Sn) into the SAM-modified layers, leading to improved passivation of defects and minimized recombination losses. This enhanced interfacial chemistry plays a pivotal role in the superior performance of SAM-modified devices. In summary, the precise manipulation of energy alignment through SAMs provides a clear trajectory toward improving both the efficiency and stability of perovskite solar cells. Future endeavors will need to evaluate long-term durability under operational conditions to ensure practical applicability.

**FIGURE 8:**
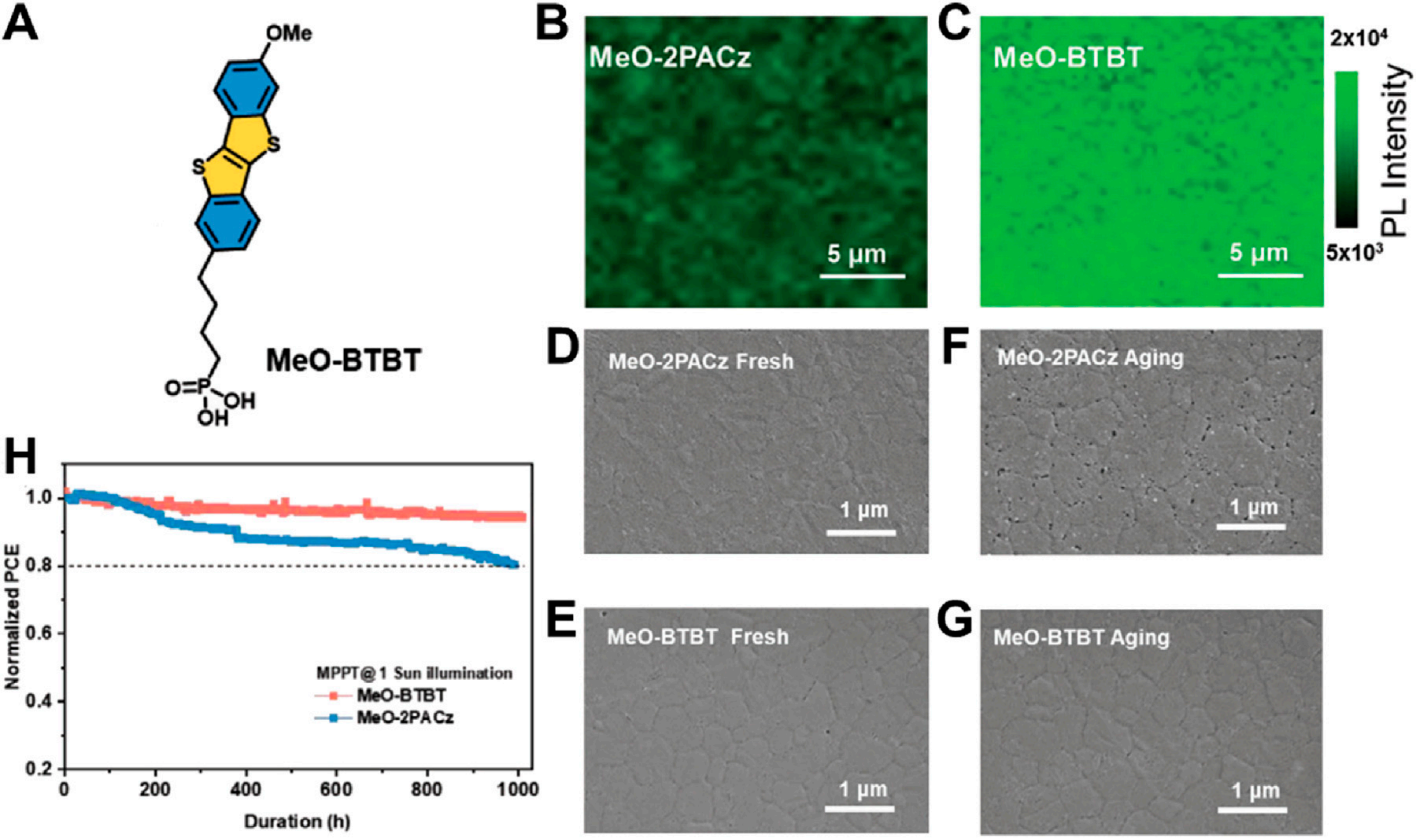
**(A)** The chemical structure of MeO-BTBT. **(B, C)** PL mapping images of perovskite films deposited on MeO-2PACz and MeO-BTBT substrates. The direction of incident light comes from the perovskite side. **(D–G)** SEM images of the fresh and aged perovskite films peeled off from the MeO-2PACz and MeO-BTBT modified substrates. **(H)** MPP tracking of PVSCs under 1 sun continuous illumination (Liu M et al., 2024).

The employment of SAMs might serve to bolster both the performance and long-term stability of perovskite solar cells. However, same anchoring groups-based SAM materials with different core might result different properties of the devices, typically for the stability. Liu et al. designed two SAM materials MeO-2PACz and MeO-BTBT as the hole selective layer to build the PVSCs (Liu M et al., 2024). Though both SAM materials contain the same anchoring groups (Figure 8A), the surface defects as well as the device performance are totally different. The author found that the significantly improved quality of perovskite was formed on the MeO-BTBT compare to the MeO-2PACz. In addition, the strong surface passivation between perovskite layer and the MeO-BTBT layer was also stronger than the ones of MeO-2PACz. This might be due to the core of the MeO-BTBT were more planar with large molecular dipole moment. The interfacial charge carrier recombination loss was investigated by the 2D PL scanning (Figures 8B, C). The results showed that the perovskite film on the MeO-2PACz presented a lower PL intensity than the ones of MeO-BTBT. This indicates that a reduction in non-radiative recombination pathways occurred for the interface of perovskite layer and the MeO-BTBT, thereby fostering improved charge transport across the interface. A comparison of the long-term operational stability of both SAMs is provided in Figure 8H, wherein MeO-BTBT-modified devices demonstrate superior stability, retaining over 94.2% of their initial power conversion efficiency after 1,000 h of continuous 1-sun illumination. Conversely, MeO-2PACz devices exhibit a more rapid decline in PCE during the same timeframe, even down to 82.1%. The totally different stability of the devices might be due to the quality of the perovskite after exposing. As shown in Figures 8D–G, the perovskite films peeled off from the various substrate exhibited totally different surface morphology after exposing. The perovskite film peeled off from MeO-BTBT presented a more compact and uniform morphology exposing over time 20 days, which is almost no change compare to the pristine film. However, there are many voids around the grain boundary for the ones of MeO-2PACz aging. In addition, the MeO-2PACz SAMs material is also not stable. It seems strong surface passivation might result the device with improved stability. Future research could investigate the scalability of MeO-BTBT-based SAMs for commercial applications.

**FIGURE 9:**
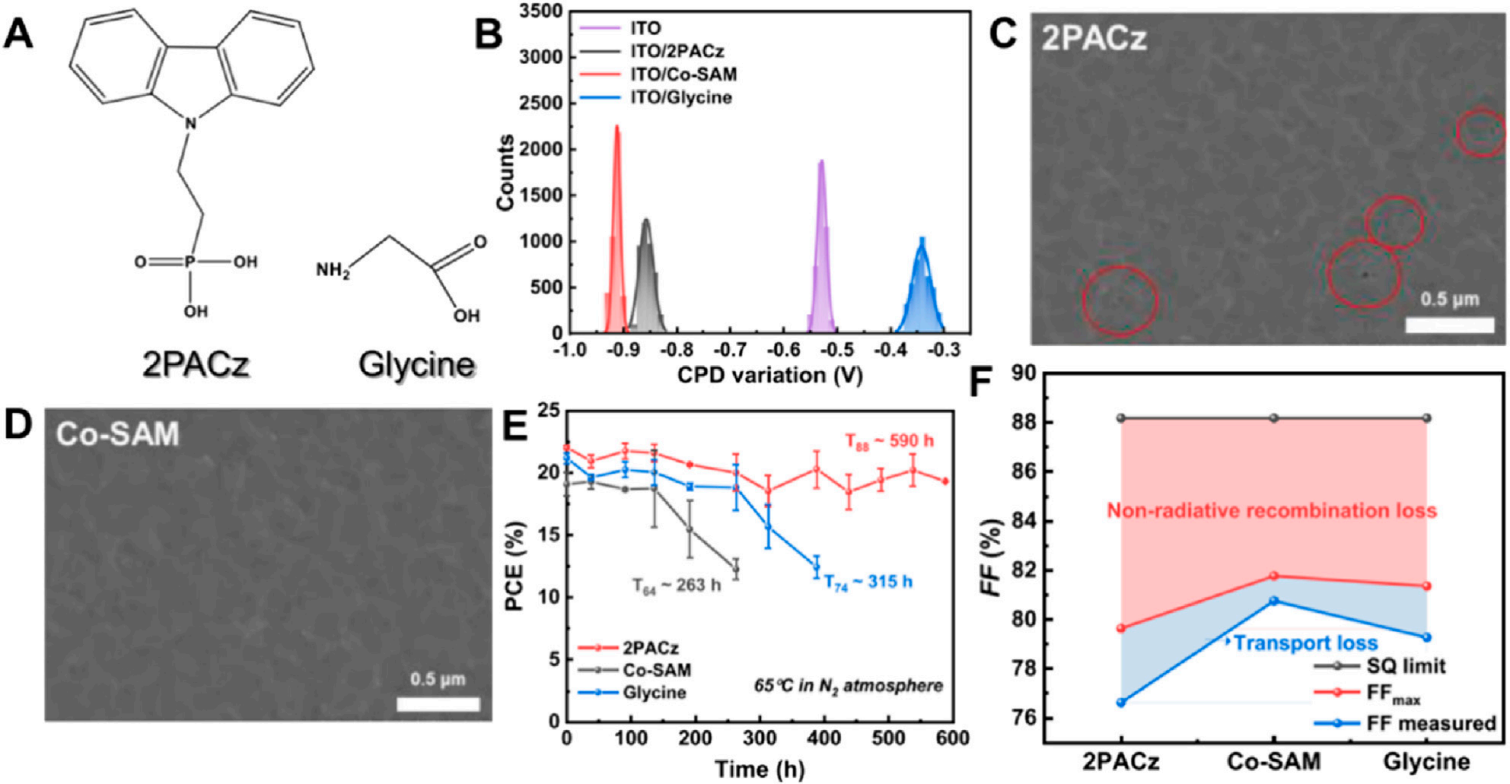
**(A)** Chemical structures of 2PACz and glycine. **(B)** The corresponding statistical contact potential difference variations obtained by KPFM. Buried interface and cross-sectional SEM images of perovskite films coated on **(C)** ITO/2PACz and **(D)** ITO/Co-SAM. **(E)** Thermal stability of unencapsulated Sn−Pb PVSCs modified by 2PACz, Co-SAM, and glycine at 65°C under dark conditions in a N2 environment. **(F)** FF loss analysis of devices with 2PACz, Co-SAM, and glycine (Roe et al., 2024).

The Co-SAM strategy, which combines 2PACz and Glycine, is designed to diminish non-radiative recombination losses while concurrently extending device longevity (Roe et al., 2024). As illustrated in Figure 9A, 2PACz and Glycine are co-adsorbed onto the ITO substrate, thereby forming a more resilient interface. Variations in contact potential difference depicted in Figure 9B indicate that Co-SAM-modified devices achieve a more stable surface potential in comparison to devices utilizing 2PACz or Glycine individually, suggesting enhanced interface passivation. Scanning electron microscopy images showcased in Figures 9C, D underscore the morphological discrepancies between Co-SAM and 2PACz-modified devices. While 2PACz films exhibit visible defects and cracks, Co-SAM films present a more uniform and flawless surface, contributing to improved charge transport and reduced recombination. In terms of performance, Figure 9E compares the power conversion efficiency over time for devices employing Co-SAM, 2PACz, and Glycine. The Co-SAM-modified devices exhibit a notably longer T80 (590 h) in contrast to both 2PACz (263 h) and Glycine (315 h), signifying enhanced stability under thermal stress at 65°C within a nitrogen atmosphere. Furthermore, the fill factor analysis presented in Figure 9F demonstrates that Co-SAM mitigates non-radiative recombination losses more effectively than the other two SAMs, bringing the FF closer to the Shockley-Queisser (SQ) limit. In conclusion, the Co-SAM approach, through its amalgamation of 2PACz and Glycine, delivers significant advancements in both device stability and performance by curbing recombination losses and bolstering interface robustness. Future research endeavors could concentrate on refining the co-adsorption process to fully maximize these benefits.

Selective contact based on molecules has become a pivotal component in ensuring the efficiency of inverted perovskite solar cells. These molecules are typically composed of a conjugated core with heteroatom substitution, aiming to achieve ideal carrier transport capabilities. To date, successful designs of conjugated cores have been limited to two *N*-substituted π-conjugated structures: carbazole and triphenylamine, with molecular optimization focusing on their derivatives. However, the improvement of device lifespan has been hindered by the inherent limitations on molecular stability induced by such heteroatom substitution structures. Robust molecular contacts that do not compromise electronic properties are urgently needed but remain a challenge.

**FIGURE 10:**
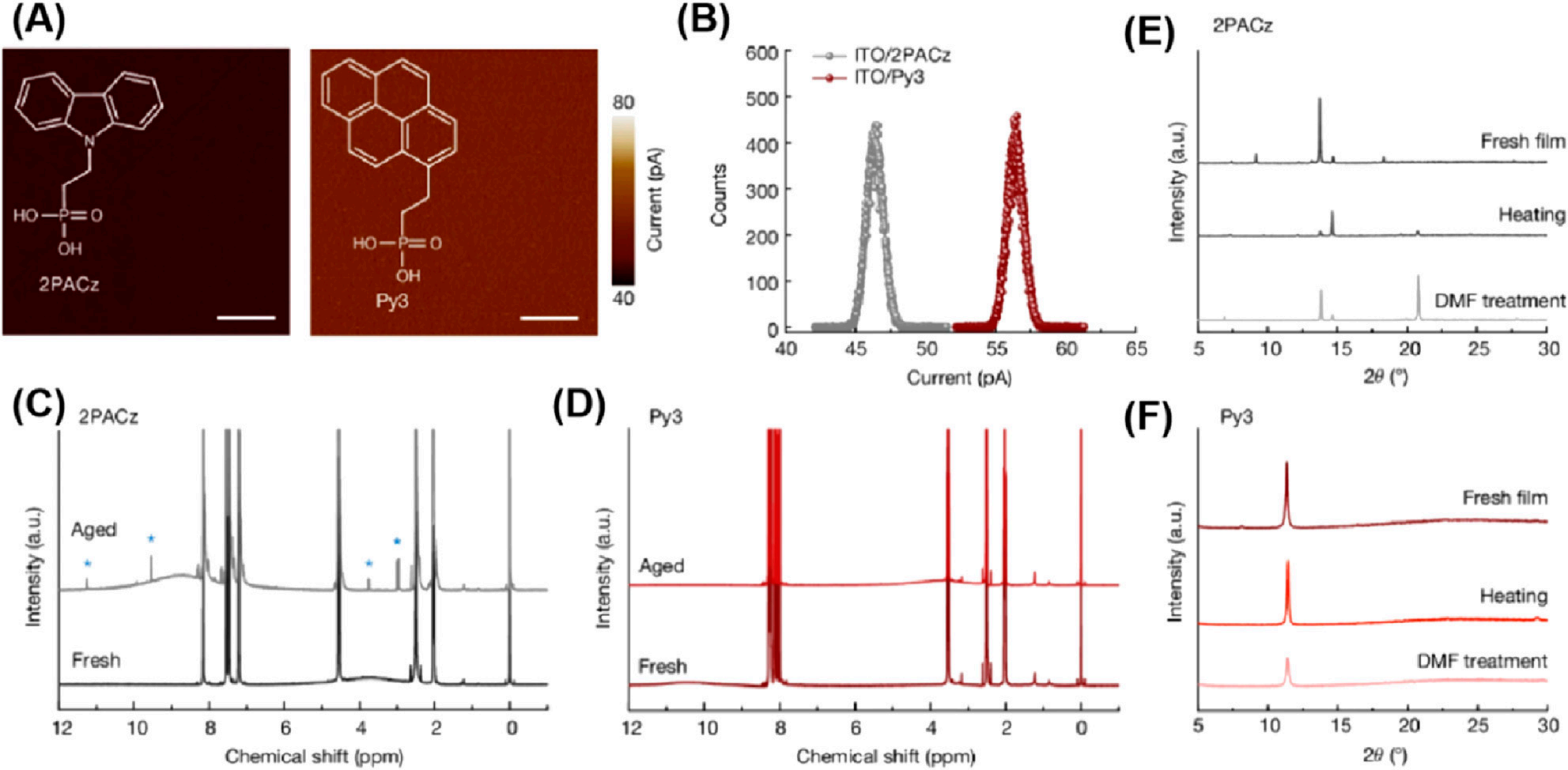
Basic properties of assembled Py3 as molecular contacts for PVSCs. c-AFM images **(A)** and the corresponding surface current signals **(B)** of the ITO substrate covered with 2PACz and with Py3. Scale bars, 2 μm. **(C, D)** NMR spectra of 2PACz **(C)** and Py3 **(D)** before and after illumination. The blue asterisks denote the new NMR peaks that emerge. **(E, F)** XRD patterns of 2PACz **(E)** and Py3 **(F)** films before and after ageing tests of thermal annealing and organic solvent treatment (Zhao et al., 2024).

In this context, Xue Jingjing and colleagues report a polycyclic aromatic hydrocarbon core structure without heteroatom substitution (Zhao et al., 2024). They designed a pyrene structure, the smallest closely fused polycyclic aromatic system, to replace the commonly used conjugated cores in the (2-(9H-carbazol-9-yl)ethyl)phosphonic acid (2PACz) or triphenylamine series. When deposited on indium tin oxide (ITO) glass substrates, Py3, like the commonly used 2PACz, provides uniform surface coverage. Figure 10A compares the conductive atomic force microscopy (c-AFM) images of ITO substrates coated with 2PACz and Py3. Although both 2PACz and Py3 exhibit uniform surface electronic properties, Py3 shows a higher average surface current signal of approximately 57 pA compared to 2PACz (about 46 pA; Figure 10B). The higher conductivity indicated by c-AFM suggests enhanced charge transport, indicating that Py3 is a promising molecular contact for facilitating charge flow despite the absence of heteroatom doping to enrich electron density. The improved charge transport capability may stem from Py3’s extended π-conjugation, which promote electron delocalization.

Due to its all-carbon aromatic skeleton, Py3 is expected to better maintain its molecular integrity under external stimuli. The researchers used nuclear magnetic resonance (NMR) spectroscopy to compare the molecular stability of 2PACz and Py3. Both molecules were aged under continuous light exposure at ambient conditions for 120 h. As shown in Figure 10C, different NMR peaks appeared in the aged 2PACz sample, indicating degradation and the formation of new chemical species. After aging, distinct peaks emerged in the alkyl (2.0–4.0 ppm) and aromatic (7.0–12.5 ppm) regions of 2PACz, suggesting N-dealkylation of the polarized C-N bonds, leading to the formation of carbazole and vinylphosphonate esters. The formed vinylphosphonate esters underwent further reactions, ultimately resulting in products containing aldehydes and carboxylic acids. In contrast, the NMR spectrum of Py3 remained nearly unchanged before and after aging (Figure 10D), confirming its significantly reduced chemical reactivity.

Beyond the inherent stability of the molecules, their structural robustness when assembled into films was also evaluated. The conjugated core always induces π-π stacking in molecules, enabling them to self-assemble into periodic structures. X-ray diffraction (XRD) measurements revealed clear diffraction patterns in both 2PACz and Py3 films, demonstrating their long-range ordered π-π assembly. The primary diffraction peak of fresh 2PACz film was located at 13.8°, accompanied by weak diffraction patterns centered at 9.2°, 14.6°, and 18.4°, indicating the presence of multiple molecular stacking modes. Upon heating, significant changes occurred in the diffraction pattern of 2PACz, with a primary peak appearing at 14.6° and the original primary peak decreasing (Figure 10E). This demonstrated that the π-π stacking structure of 2PACz molecules had changed, indicating instability of the stacking mode under external thermal stress. In contrast, a diffraction peak appeared in the fresh Py3 film and remained almost unchanged after heating, suggesting a strong and dominant stacking mode in Py3 (Figure 10F).

When the perovskite layer was subsequently deposited on these molecular layers to construct devices, their resistance to dimethylformamide (DMF), a commonly used organic solvent for perovskite layer deposition, was further tested. Similar to the thermal test, the XRD peak position of Py3 remained at 11.4° after DMF treatment, while different diffraction patterns appeared in the 2PACz film, indicating structural deformation after DMF treatment (Figures 10E, F). The enhanced resistance of Py3 films to DMF helps minimize structural deformation at the interface during the perovskite film deposition process. This is beneficial for interface contact between molecules and perovskite, thereby suppressing non-radiative carrier recombination. This core structure produces relatively chemically inert and structurally rigid molecular contacts, significantly improving the performance of perovskite solar cells in terms of efficiency and durability. Under various accelerated aging tests, the device efficiency reached up to 26.1% (certified by a third-party institution as 25.7%), with a substantial increase in lifespan.

**FIGURE 11:**
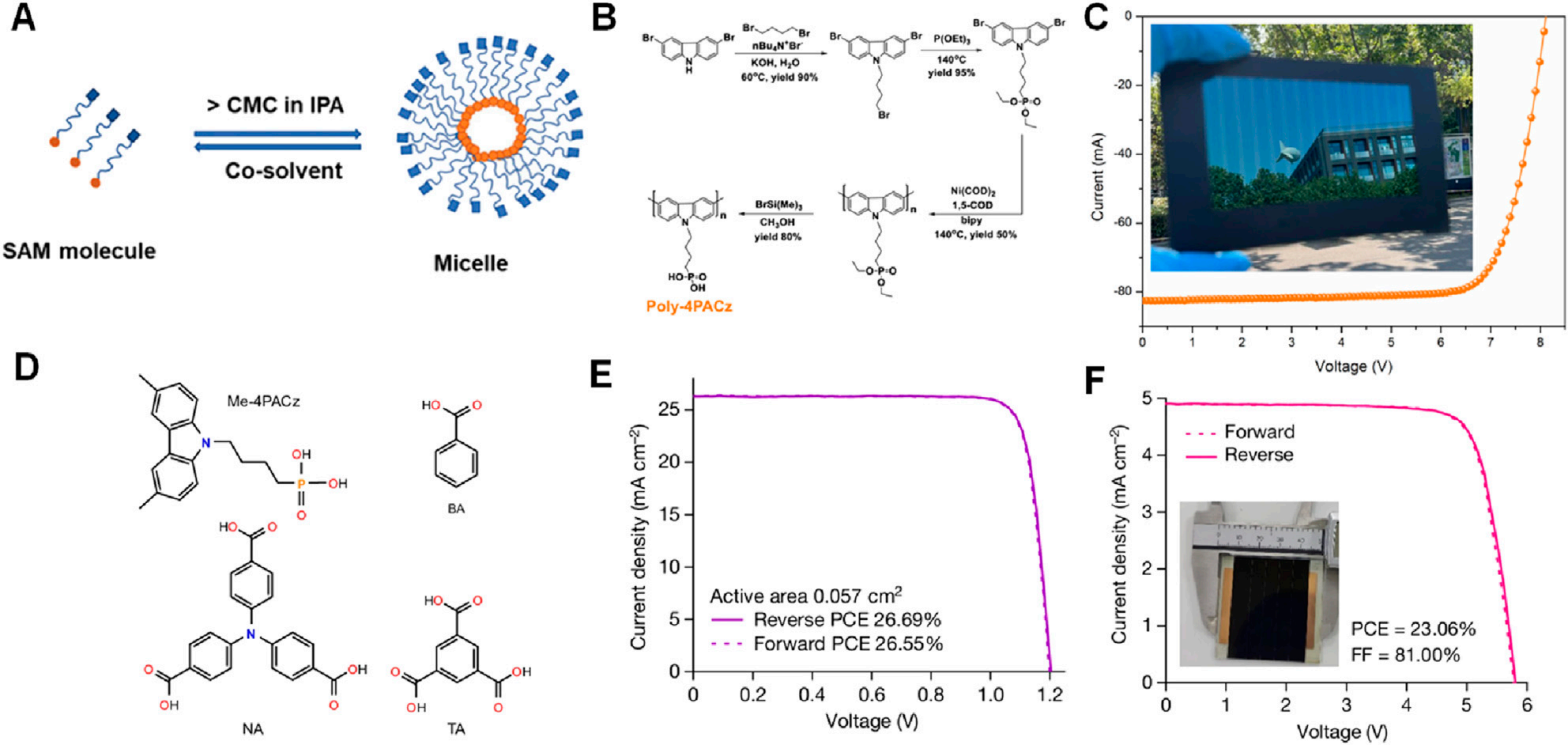
**(A)** Illustration of micelles formed from the amphiphilic SAM molecules anddisassembled in co-solvent (Liu M et al., 2023). **(B)** Synthesis route of Poly-4PACz. **(C)** *J*-*V* characteristics of the Poly-4PACz-based perovskite mini-module with an aperture area of 25.0 cm^2^. Inset shows a picture of Poly-4PACz-based perovskite module (Ren et al., 2023). **(D)** before and after illumination. The blue asterisks denote the new NMR peaks that emerge. e,f, XRD patterns of 2PACz **(E)** and Py3 **(F)** films before and after ageing tests of thermal annealing and organic solvent treatment (Liu S et al., 2024).

It is noteworthy that most of the hole-selective SAMs are inherently amphipathic, (Zhang et al., 2023) thus these SAMs tend to form micellar nanoparticles in solution (Figure 11A), (Liu L et al., 2023) and the aggregation phenomenon of SAM molecules on self-adsorbed materials is very serious, which is extremely fatal for solar cells. This also leads to poorer performance in terms of scalability and stability for these devices. The Chen’s team designed and synthesized a novel type of HTM (hole transport material) through the polymerization of carbazole phosphoric acid small molecules, resulting in Poly-4PACz (Figure 11B) (Ren et al., 2023). Compared to the currently popular small molecule PACz HTM, Poly-4PACz possesses several key advantages: excellent hole extraction capability, high electrical conductivity, and good wetting properties. Poly-4PACz can be uniformly coated on large ITO substrates, and when coated on ITO module substrates covered with Poly-4PACz, large perovskite films exhibit a mirror-like surface (as shown in the inset of Figure 4B). Perovskite modules based on Poly-4PACz can achieve an impressive 20.7% PCE on a cell area of 25.0 cm^2^ (Figure 11C), with *V*_OC_ of 8.11 V (1.16 V per sub-cell), short-circuit current (ISC) of 82.5 mA, FF of 0.773. Considering a geometric FF (GFF) of 92%, the aperture area efficiency corresponds to an effective area PCE of 22.5% (as shown in Figure 11C). Overall, these results indicate that this polymer material is fully compatible with the scalable manufacturing of perovskite modules, and Poly-4PACz has a promising application in the actual manufacturing of perovskite photovoltaics.

The Chen’s team reported the molecular hybridization at the inverted PVSK buried interface, which combined the popular self-assembled molecule [4-({3,6-bis(3,6,9-trimethyl-9H-carbazol-9-yl)propyl}amino)butyl]phosphonic acid (Me-4PACz) with multiple aromatic carboxylic acids, 4,4′,4″-tris(aminotriazine)phenol (NA) (Figure 11D), to improve the heterojunction interface (Liu S et al., 2024). The molecular hybridization of Me-4PACz with NA significantly improved the interface characteristics. The resulting inverted perovskite solar cells exhibited a record certified steady-state efficiency of 26.54% (Figure 11E). Importantly, this strategy seamlessly integrates with large-scale manufacturing, achieving one of the highest certified power conversion efficiencies for inverted mini-modules at 22.74% (with an aperture area of 11.1 cm^2^, Figure 11F).

**FIGURE 12:**
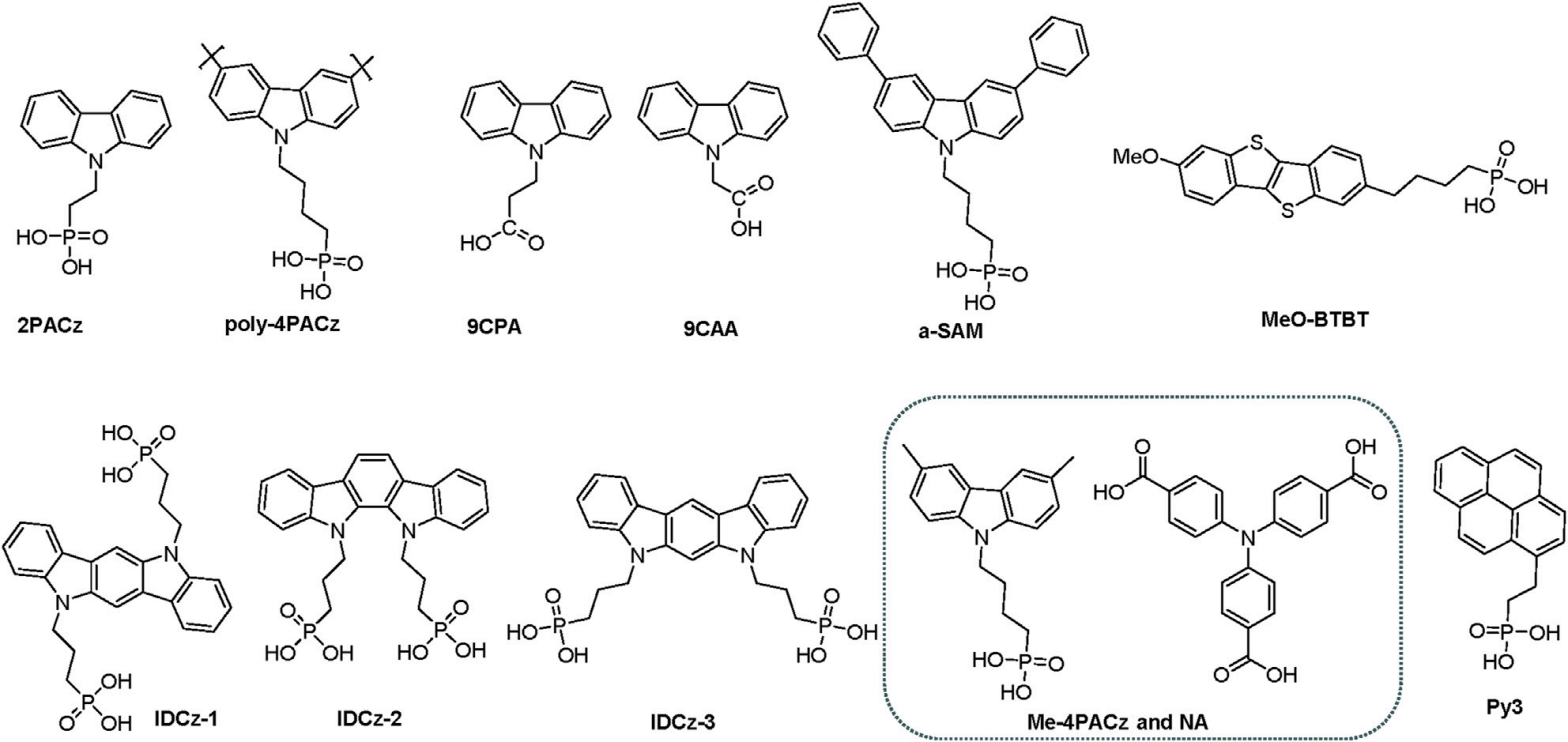
Summarized the most representative variety of SAMs in recent years.

Long-term operational stability research is necessary when subjected to environmental stressors, such as exposure to moisture and fluctuations in temperature (Jiang et al., 2023). Li et al. research reveal that to composite the SAMs with NiOx to the nanoparticle as the hole selective layer can significantly improve the device stability to the high temperature (Li Z et al., 2023). This might be due to that introducing the NiOx to the SAMs particles can form a more contact and binding between the hole selective layer and the perovskite as well as the ITO, which promote a strong interface toughening effects under thermal stress. The PCE of the PVSCs has almost comparable with the silicon-based solar cells. Thus, the device stability plays a key role to promote the PVSCs commercialization. Recently research revised that introducing SAMs as the hole selective layer could not only enhance the PCE of the device, but also improve its stability. The chemical structures of most representative variety of SAMs in recent years as well as their corresponding device stability are summarized in Figure 12 and Table 1. Further modification the chemical structures to obtain the high-performance SAMs is always a challenging, which should be focused on: (i) the stability of the SAMs materials; (ii) development novel and useful anchoring groups which might be formation strong binding and a good contact to against the external stress; (iii) interface engineering between the SAMS and the ITOs as well as the perovskite layer; (iv) effective conjugation system of the SAM’s core.

**TABLE 1:** Stability of SAMs based PVCs.

Compound	Measure condition 1	Stability	Measure condition 2	Stability	Ref.
2PACz	2500 h, N_2,_ atmosphere	85%	500 h, MPP under AM 1.5G, 23–26°C, RH: 20%–40%	85%	Adv. Mater. 2024, 36, 2312264
9CAA		93%		89%	
9CPA		89%		87%	
4PACZ	1,200 h, 1-sun, N_2,_65°C	77%			Angew. Chem. 2024, e202411730
CBzPPA		53%			
CbzNaphPPA		94%			
MeO-2PACz	1,000 h, N_2,_65°C	82.3%			Adv. Energy Mater. 2024, 14, 2303742
MeO-BTBT		93.5%			
NIO_X_/2PACz	1,400 h, unencapsulated, N_2,_ 25°C	52%	1,400 h, unencapsulated, air, 25°C, RH: 50%–75%	26%	Adv. Funct. Mater. 2024, 34, 2316202
NIO_X_/2PACz/KF		90%		80%	
4PACZ	1,000 h, unencapsulated, dark, 60°C ± 5°C, RH: 50% ± 5%	53%			Angew. Chem.Int. Ed. 2024, e202416188
K1		66%			
KF		75%			
IDCz-1	1800 h, unencapsulated, N_2,_ RT	76%			Adv. Mater. 2024, 36, 2401537
IDCz-2		89%			
IDCz-3		98%			
SAM	1,000 h, 85°C and 85% relative humidity	93.6%	1000 h, MPP 1 sun	97.3%	Adv. Energy Mater. 2024, 14, 2401303
PTAA		85.7%		89.2%	
a- SAM	400 h, encapsulated MPP, RH 85%	99%	1,000 h, encapsulated, dark, N_2_, 85°C	90%	Nat. Photonics, doi: org/10/1038/s41566-024-01531-x
c-SAM		84%		56%	
CA	600 h, unencapsulated, N_2_, 65°C	92%	1,000 h, MPP, ambient atmosphere	90%	Nat. Commun. (2024) 15:7605
NiO_x_/Me-4PACz	1000 h, MPP 1-sun 55°C	88%			Adv. Mater. 2024, 36, 2311970
NiO_x_/Me-4PACz/PC		93%			
DC-TMPS /ALD ITD	1,000 h, dark, 85°C RH 85%	98.9%	2000 h, encapsulation, MPP under simulated AM1.5G radiation, 85°C	98.5%	Science, 2024, 383, 1,236–1,240
MeO-2PACz/ALD ITD		95%		93.6%	
MeO-2PACz/ITD		61.7%		63.2%	
2PACZ	1,680 h, dark, N_2,_ RT	87.6%	263 h, dark, N_2_, 65°C	64%	ACS Nano 2024,18,24306–24316
Co-SAM		91.8%	590 h, dark, N_2_, 65°C	88%	
Glycine		91.6%	315 h, dark, N_2_, 65°C	74%	
2PACz	850 h MPP 1-sun 35°C	80%	600 h MPP 1-sun 55°C	45%	Nature 2024 doi:org/10.1038/s41586-024-07712–6
Py3		99.5%		99%	
Poly-4PACz	1500 h MPP AM1.5G	94%			Joule 2023,12,20,7, 2894–2904
NA	500 h 85°C and 85% relative humidity	99.8%	2500 h Encapsulated devices MPP 1-sun 65°C	96.1%	Nature 2024 632, 536-544

## 2 Conclusion

Self-assembled monolayers (SAMs) have emerged as a powerful tool in the interface engineering of perovskite solar cells, capable of precisely tuning energy level alignment, enhancing carrier extraction, and improving long-term device stability (Figure 12; Table 1). Through various molecular designs, including the use of different anchoring groups, coadsorption strategies, and the introduction of homogeneity, SAMs have shown great potential in addressing critical challenges such as defect passivation and non-radiative recombination losses. In this review, we explore the progress of strategies based on SAMs, starting with 2PACz, which provides foundational insights into energy level optimization and performance improvement. We then introduce coadsorbed SAMs such as the combination of 2PACz and PyCA-3F, which further improves interface passivation and reduces carrier recombination. Additionally, regulating the molecular arrangement in both crystalline and amorphous SAMs, as well as coassembling 2PACz with glycine, has shown further improvements in carrier transport efficiency and device stability. We emphasize innovative materials such as non-carcinogenic multi-aromatic MeO-BTBT and fully carbonaceous Py3, which can significantly enhance device lifetime and operational performance. Furthermore, we summarize strategies for modifying SAMs from small to large-area modules: Poly-SAM and the introduction of carboxylic acid into the Me-4PACz layer to enhance the wetting of perovskite precursors on NiO/SAM substrates. In addition, although the interface engineering based on SAMs has made significant progress in improving the efficiency and stability of perovskite solar cells, there are still challenges. It is necessary to further explore long-term operational stability under real-world conditions, such as moisture and temperature fluctuations. From the perspectives of high performance, stability, and large-area scalability, we believe that the development of SAMs will move towards the direction of polymeric full-carbon SAMs, attracting more researchers to join due to the selection of suitable energy level conjugated modules. In summary, SAMs represent a flexible and promising approach for advancing the PVSK technology. Continuous innovation in molecular design, coadsorption technologies, and stability assessments is crucial for breaking through the performance limits of the devices and accelerating the pace towards commercialization.

## References

[B1] Aktas E. Jiménez-López J. Azizi K. Torres T. Palomares E. (2020). Self-assembled Zn phthalocyanine as a robust p-type selective contact in perovskite solar cells. Nanoscale Horiz. 5 (10), 1415–1419. 10.1039/d0nh00443j 32856637

[B2] Artiom M. Amran Al-Ashouri E. K. Simona S. Gediminas N. Niaura G. Jošt M. (2018). Self-assembled hole transporting monolayer for highly efficient perovskite solar cells. Adv. Energy Mat. 8, 1801892. 10.1002/aenm.201801892

[B3] Aydin E. Ugur E. Yildirim B. K. Allen T. G. Dally P. Razzaq A. (2023). Enhanced optoelectronic coupling for perovskite/silicon tandem solar cells. Nature 623 (7988), 732–738. 10.1038/s41586-023-06667-4 37769785

[B4] Cao Q. Wang T. Pu X. He X. Xiao M. Chen H. (2024). Co‐Self‐Assembled monolayers modified NiO_x_ for stable inverted perovskite solar cells. Adv. Mat. 36, 2311970. 10.1002/adma.202311970 38198824

[B5] Caprioglio P. Smith J. A. Oliver R. D. J. Dasgupta A. Choudhary S. Farrar M. D. (2023). Open-circuit and short-circuit loss management in wide-gap perovskite p-i-n solar cells. Nat. Commun. 14 (1), 932. 10.1038/s41467-023-36141-8 36805448 PMC9941504

[B6] Cassella E. J. Spooner E. L. K. Thornber T. O'Kane M. E. Catley T. E. Bishop J. E. (2022). Gas-assisted spray coating of perovskite solar cells incorporating sprayed self-assembled monolayers. Adv. Sci. 9, e2104848. 10.1002/advs.202104848 PMC9108661 35142096

[B7] Chang C. Y. Huang H. H. Tsai H. Lin S. L. Liu P. H. Chen W. (2021). Facile fabrication of self-assembly functionalized polythiophene hole transporting layer for high performance perovskite solar cells. Adv. Sci. 8 (5), 2002718. 10.1002/advs.202002718 PMC7927620 33717841

[B8] Dai Z. Li S. Liu X. Chen M. Athanasiou C. E. Sheldon B. W. (2022). Dual-interface-reinforced flexible perovskite solar cells for enhanced performance and mechanical reliability. Adv. Mat. 34 (47), e2205301. 10.1002/adma.202205301 36148590

[B9] Dai Z. Yadavalli S. K. Chen M. Abbaspourtamijani A. Qi Y. Padture N. P. (2021). Interfacial toughening with self-assembled monolayers enhances perovskite solar cell reliability. Science 372 (6542), 618–622. 10.1126/science.abf5602 33958474

[B10] Deng X. Qi F. Li F. Wu S. Lin F. R. Zhang Z. (2022). Co-Assembled monolayers as hole-selective contact for high-performance inverted perovskite solar cells with optimized recombination loss and long-term stability. Angew. Chem. Int. Ed. Engl. 61 (30), e202203088. 10.1002/anie.202203088 35560775

[B11] Ernestas K. Marius F. Simonas D. Vidmantas G. (2024). Charge carrier dynamics at the perovskite interface with self-assembled monolayers. ACS Appl. Mat. Interface 18, 39423047. 10.1021/acsami.4c10223 PMC11533155 39423047

[B12] Farag A. Feeney T. Hossain I. Schackmar F. Fassl P. Küster K. (2023). Evaporated self-assembled monolayer hole transport layers: lossless interfaces in p-i-n perovskite solar cells. Adv. Energy Mat. 13, 2203982. 10.1002/aenm.202203982

[B13] Feleki B. T. Bouwer R. K. M. Zardetto V. Wienk M. M. Janssen R. A. J. (2022). p-i-n perovskite solar cells on steel substrates. ACS Appl. Energy. Mat. 5 (6), 6709–6715. 10.1021/acsaem.2c00291 PMC9241001 35783346

[B14] Galvis C. E. P. González Ruiz D. A. Martínez-Ferrero E. Palomares E. (2023). Challenges in the design and synthesis of self-assembling molecules as selective contacts in perovskite solar cells. Chem. Sci. 15 (5), 1534–1556. 10.1039/d3sc04668k 38303950 PMC10829004

[B15] Gao M. Xu X. Tian H. Ran P. Jia Z. Su Y. (2024). Enhancing efficiency of large-area wide-bandgap perovskite solar modules with spontaneously formed self-assembled monolayer interfaces. J. Phys. Chem. Lett. 15 (15), 4015–4023. 10.1021/acs.jpclett.4c00814 38577843

[B16] Han P. Zhang Y. (2024). Recent advances in carbazole-based self-assembled monolayer for solution-processed optoelectronic devices. Adv. Mater 36 (33), e2405630. 10.1002/adma.202405630 38940073

[B17] He R. Wang W. Yi Z. Lang F. Chen C. Luo J. (2023). Improving interface quality for 1-cm2 all-perovskite tandem solar cells. Nature 618 (7963), 80–86. 10.1038/s41586-023-05992-y 36990110

[B18] Hou M. Zhang H. Wang Z. Xia Y. Chen Y. Huang W. (2018). Enhancing efficiency and stability of perovskite solar cells via a self-assembled dopamine interfacial layer. ACS Appl. Mat. Interfaces 10 (36), 30607–30613. 10.1021/acsami.8b10332 30118201

[B19] Hsu H. C. Tsao J. C. Yeh C. H. Wu H. T. Wu C. T. Wu S. H. (2024). Large-area perovskite solar module produced by introducing self-assembled L-histidine monolayer at TiO2 and perovskite interface. Nanomaterials 14 (15), 1315. 10.3390/nano14151315 39120420 PMC11314024

[B20] Huang Y. Tao M. Zhang Y. Wang Z. Sun Z. Zhang W. (2024). Asymmetric modification of carbazole based self-assembled monolayers by hybrid strategy for inverted perovskite solar cells. Angew. Chem. Int. Ed. Engl. 5, e202416188. 10.1002/anie.202416188 39367792

[B21] Hung C. M. Mai C. L. Wu C. C. Chen B. H. Lu C. H. Chu C. C. (2023). Self-assembled monolayers of Bi-functionalized porphyrins: a novel class of hole-layer-coordinating perovskites and indium tin oxide in inverted solar cells. Angew. Chem. Int. Ed. Engl. 62 (40), e202309831. 10.1002/anie.202309831 37594921

[B22] Jiang Q. Tirawat R. Kerner R. A. Gaulding E. A. Xian Y. Wang X. (2023). Towards linking lab and field lifetimes of perovskite solar cells. Nature 623 (7986), 313–318. 10.1038/s41586-023-06610-7 37696288

[B23] Jiang W. Li F. Li M. Qi F. Lin F. R. Jen A. K. (2022). π-Expanded carbazoles as hole-selective self-assembled monolayers for high-performance perovskite solar cells. Angew. Chem. Int. Ed. Engl. 61, e202213560. 10.1002/anie.202213560 36300589

[B24] Jiang W. Liu M. L i Y. Lin F. R. Jen A. K. (2024a). Rational molecular design of multifunctional self-assembled monolayers for efficient hole selection and buried interface passivation in inverted perovskite solar cells. Chem. Sci. 15 (8), 2778–2785. 10.1039/d3sc05485c 38404377 PMC10882494

[B25] Jiang W. Wang D. Shang W. Li Y. Zeng J. Zhu P. (2024b). Spin-coated and vacuum-processed hole-extracting self-assembled multilayers with H-aggregation for high-performance inverted perovskite solar cells. Angew. Chem. Int. Ed. Engl. 63 (45), e202411730. 10.1002/anie.202411730 39044319

[B26] Kim G. Kim H. Kim M. Sin J. Kim M. Kim J. (2024). Enhancing surface modification and carrier extraction in inverted perovskite solar cells via self-assembled monolayers. Nanomaterials 14 (2), 214. 10.3390/nano14020214 38276732 PMC10821478

[B27] Li B. Zhang C. Gao D. Sun X. Zhang S. Li Z. (2024). Suppressing oxidation at perovskite–NiO_x_ interface for efficient and stable tin perovskite solar cells. Adv. Mat. 36 (17), e2309768. 10.1002/adma.202309768 37971969

[B28] Li D. Lian Q. Du T. Ma R. Liu H. Liang Q. (2024). Co-adsorbed self-assembled monolayer enables high-performance perovskite and organic solar cells. Nat. Commun. 15, 7605. 10.1038/s41467-024-51760-5 39218952 PMC11366757

[B29] Li M. Li Z. Liu M. Fu H. Qi F. Lin F. R. (2024). A hole-selective self-assembled monolayer for both efficient perovskite and organic solar cells. Langmuir 40 (9), 4772–4778. 10.1021/acs.langmuir.3c03610 38381871

[B30] Li W. Cariello M. Méndez M. Cooke G. Palomares E. (2023). Self-assembled molecules for hole-selective electrodes in highly stable and efficient inverted perovskite solar cells with ultralow energy loss. ACS Appl. Energy. Mat. 6 (3), 1239–1247. 10.1021/acsaem.2c02880 PMC9930087 36817750

[B31] Li Z. Sun X. Zheng X. Li B. Gao D. Zhang S. (2023). Stabilized hole-selective layer for high-performance inverted p-i-n perovskite solar cells. Science 382, 284–289. 10.1126/science.ade9637 37856581

[B32] Liao Q. Wang Y. Hao M. Li B. Yang K. Ji X. (2022a). Green-solvent-processable low-cost fluorinated hole contacts with optimized buried interface for highly efficient perovskite solar cells. ACS Appl. Mat. Interfaces 14, 43547–43557. 10.1021/acsami.2c10758 36112992

[B33] Liao Q. Wang Y. Zhang Z. Yang K. Shi Y. Feng K. (2022b). Self-assembled donor-acceptor hole contacts for inverted perovskite solar cells with an efficiency approaching 22%: the impact of anchoring groups. J. Energy. Chem. 68, 87–95. 10.1016/j.jechem.2021.11.001

[B34] Lin P. A. Yang B. Lin C. Fan Z. Chen Y. Zhang W. (2024). A regulation strategy of self-assembly molecules for achieving efficient inverted perovskite solar cells. Phys. Chem. Chem. Phys. 26 (19), 14305–14316. 10.1039/d4cp00509k 38693910

[B35] Lin X. Jumabekov A. N. Lal N. N. Pascoe A. R. Gómez D. E. Duffy N. W. (2017). Dipole-field-assisted charge extraction in metal-perovskite-metal back-contact solar cells. Nat. Commun. 8 (1), 613. 10.1038/s41467-017-00588-3 28931833 PMC5606993

[B36] Liu L. Mei A. Liu T. Jiang P. Sheng Y. Zhang L. (2015). Fully printable mesoscopic perovskite solar cells with organic silane self-assembled monolayer. J. Am. Chem. Soc. 137 (5), 1790–1793. 10.1021/ja5125594 25594109

[B37] Liu L. Yang Y. Du M. Cao Y. Ren X. Zhang L. (2023). Self-assembled amphiphilic monolayer for efficient and stable wide-bandgap perovskite solar cells. Adv. Energy Mat. 13, 2202802. 10.1002/aenm.202202802

[B38] Liu M. Bi L. Jiang W. Zeng Z. Tsang S. W. Lin F. R. (2023). Compact hole-selective self-assembled monolayers enabled by disassembling micelles in solution for efficient perovskite solar cells. Adv. Mat. 35 (46), 2304415. 10.1002/adma.202304415 37487572

[B39] Liu M. Li M. Li Y. An Y. Yao Z. Fan B. (2024). Defect-passivating and stable benzothiophene-based self-assembled monolayer for high-performance inverted perovskite solar cells. Adv. Energy Mat. 14, 2303742. 10.1002/aenm.202303742

[B40] Liu S. Li J. X. W. Chen R. Sun Z. Zhang Y. (2024). Buried interface molecular hybrid for inverted perovskite solar cells. Nature 632, 536–544. 10.1038/s41586-024-07723-3 38925147

[B41] Marchant C. Williams R. M. (2024). Perovskite/silicon tandem solar cells-compositions for improved stability and power conversion efficiency. Photochem. Photobiol. Sci. 23 (1), 1–22. 10.1007/s43630-023-00500-7 37991706

[B42] Park S. M. Wei M. Lempesis N. Yu W. Hossain T. Agosta L. (2023). Low-loss contacts on textured substrates for inverted perovskite solar cells. Nature 624 (7991), 289–294. 10.1038/s41586-023-06745-7 37871614

[B43] Phung N. Verheijen M. Todinova A. Datta K. Verhage M. Al-Ashouri A. (2022). Enhanced self-assembled monolayer surface coverage by ALD NiO in p-i-n perovskite solar cells. Acs. Appl. Mater Interfaces 14 (1), 2166–2176. 10.1021/acsami.1c15860 34936322 PMC8763377

[B44] Ren Z. Cui Z. Shi X. Wang L. Dou Y. Wang F. (2023). Poly(carbazole phosphonic acid) as a versatile hole-transporting material for p-i-n perovskite solar cells and modules. Joule 7, 2894–2904. 10.1016/j.joule.2023.10.014

[B45] Roe J. Son J. Park S. Seo J. Song T. Kim J. (2024). Synergistic buried interface regulation of Tin−Lead perovskite solar cells via Co-self assembled monolayers. Acs. Nano. 18, 24306–24316. 10.1021/acsnano.4c06396 39172688

[B46] Sekimoto T. Yamamoto T. Takeno F. Nishikubo R. Hiraoka M. Uchida R. (2023). Perovskite solar cell using isonicotinic acid as a gap-filling self-assembled monolayer with high photovoltaic performance and light stability. ACS Appl. Mat. Interfaces 15 (28), 33581–33592. 10.1021/acsami.3c05215 37417321

[B47] Seo Y. K. Cho S. J. Seo E. B. He X. Yoon H. J. (2020). Self-assembled monolayers as interface engineering nanomaterials in perovskite solar cells. Adv. Energy Mat. 10, 2002606. 10.1002/aenm.202002606

[B48] Shen X. Gallant B. M. Holzhey P. Smith J. A. Elmestekawy K. A. Yuan Z. (2023). Chloride-based additive engineering for efficient and stable wide-bandgap perovskite solar cells. Adv. Mat. 35 (30), e2211742. 10.1002/adma.202211742 37191054

[B49] Sun X. Zhu Z. Li Z. (2022). Recent advances in developing high-performance organic hole transporting materials for inverted perovskite solar cells. Front. Optoelectron. 15 (1), 46. 10.1007/s12200-022-00050-3 36637605 PMC9756258

[B50] Sun Y. Lai Y. Yang Y. M. (2024). Progress of hole-transport layers in mixed Sn-Pb perovskite solar cells. Small 20, e2406991. 10.1002/smll.202406991 39324229

[B51] Tang H. Shen Z. Shen Y. Yan G. Wang Y. Han Q. (2024). Reinforcing self-assembly of hole transport molecules for stable inverted perovskite solar cells. Science 383 (6688), 1236–1240. 10.1126/science.adj9602 38484063

[B52] Tong X. Xie L. Li J. Pu Z. Du S. Yang M. (2024). Large orientation angle buried substrate enables efficient flexible perovskite solar cells and modules. Adv. Mat. 36 (38), e2407032. 10.1002/adma.202407032 39049807

[B53] Valles-Pelarda M. Hames B. C. García-Benito I. Almora O. Molina-Ontoria A. Sánchez R. S. (2016). Analysis of the hysteresis behavior of perovskite solar cells with interfacial fullerene self-assembled monolayers. J. Phys. Chem. Lett. 7 (22), 4622–4628. 10.1021/acs.jpclett.6b02103 27797214

[B54] Wang J. Liu N. Liu Z. Liu J. Zhou C. Zhang J. (2024). Versatile self-assembled monolayer enables high-performance inverted CsPbI3 perovskite solar cells. ACS Appl. Nano Mat. 7 (13), 15267–15276. 10.1021/acsanm.4c02064

[B55] Wang X. Li J. Guo R. Yin X. Luo R. Guo D. (2024). Regulating phase homogeneity by self-assembled molecules for enhanced efficiency and stability of inverted perovskite solar cells. Nat. Photonics 18, 1269–1275. 10.1038/s41566-024-01531-x

[B56] Wang Y. Liao Q. Chen J. Huang W. Zhuang X. Tang Y. (2020). Teaching an old anchoring group new tricks: enabling low-cost, eco-friendly hole-transporting materials for efficient and stable perovskite solar cells. J. Am. Chem. Soc. 142, 16632–16643. 10.1021/jacs.0c06373 32852200

[B57] Wojciechowski K. Stranks S. D. Abate A. Sadoughi G. Sadhanala A. Kopidakis N. (2014). Heterojunction modification for highly efficient organic-inorganic perovskite solar cells. ACS Nano 8 (12), 12701–12709. 10.1021/nn505723h 25415931

[B58] Wolff C. M. Canil L. Rehermann C. Ngoc Linh N. Zu F. (2020). Perfluorinated self-assembled monolayers enhance the stability and efficiency of inverted perovskite solar cells. ACS Nano 14 (2), 1445–1456. 10.1021/acsnano.9b03268 31909973

[B59] Wu J. Yan P. Yang D. Guan H. Yang S. Cao X. (2024). Bisphosphonate-anchored self-assembled molecules with larger dipole moments for efficient inverted perovskite solar cells with excellent stability. Adv. Mat. 36, 2401537. 10.1002/adma.202401537 38768481

[B60] Wu Y. Song J. Wu X. Qiu C. Yin X. Hu L. (2022). Highly efficient and stable ZnO-based perovskite solar cells enabled by a self-assembled monolayer as the interface linker. Chem. Commun. 58 (66), 9266–9269. 10.1039/d2cc03890k 35903987

[B61] Xu Z. Wang J. Liu Z. Xiao C. Wang J. Liu N. (2024). Self-assembled monolayer suppresses interfacial reaction between NiO_x_ and perovskite for efficient and stable inverted inorganic perovskite solar cells. ACS Appl. Mater Interfaces 16 (40), 53811–53821. 10.1021/acsami.4c11177 39318177

[B62] Yao Y. Cheng C. Zhang C. Hu H. Wang K. De Wolf S. (2022). Organic hole-transport layers for efficient, stable, and scalable inverted perovskite solar cells. Adv. Mat. 34 (44), e2203794. 10.1002/adma.202203794 35771986

[B63] Yeo D. Shin J. Kim D. Jaung J. Y. Jung I. H. (2024). Self-assembled monolayer-based hole-transporting materials for perovskite solar cells. Nanomaterials 14 (2), 175. 10.3390/nano14020175 38251141 PMC10818599

[B64] Zhang H. Pfeifer L. Zakeeruddin S. M. Chu J. Grätzel M. (2023). Tailoring passivators for highly efficient and stable perovskite solar cells. Nat. Rev. Chem. 7, 632–652. 10.1038/s41570-023-00510-0 37464018

[B71] Zhang S. Ye F. Wang X. Chen R. Zhang H. Zhan J. (2023). Minmizing buried interfacial defects for efficient inverted perovskite solar cells. Science 38, 404–409. 10.1126/science.adg3755 37104579

[B65] Zhang Z. Zhu R. Tang Y. Su Z. Hu F. Zhang H. (2024). Anchoring charge selective self-assembled monolayers for tin-lead perovskite solar cells. Adv. Mat. 36, 2312264. 10.1002/adma.202312264 38281081

[B66] Zhao K. Liu Q. Yao L. Değer C. Shen J. Zhang X. (2024). peri-Fused polyaromatic molecular contacts for perovskite solar cells. Nature 632, 301–306. 10.1038/s41586-024-07712-6 39048825

[B67] Zhong L. Liu C. Lai S. Li B. Zheng B. Zhang X. (2024). Recent advances in self-assembled molecular application in solar cells. Nanomaterials 14 (9), 779. 10.3390/nano14090779 38727372 PMC11085869

[B68] Zhu J. Luo Y. He R. Chen C. Wang Y. Luo J. (2022). A donor–acceptor-type hole-selective contact reducing non-radiative recombination losses in both subcells towards efficient all-perovskite tandems. Nat. Energy. 8, 714–724. 10.1038/s41560-023-01274-z

[B69] Zhu T. Su J. Labat F. Ciofini I. Pauporté T. (2020). Interfacial engineering through chloride-functionalized self-assembled monolayers for high-performance perovskite solar cells. ACS Appl. Mater Interfaces 12 (1), 744–752. 10.1021/acsami.9b18034 31813217

[B70] Zuo L. Chen Q. De Marco N. Hsieh Y. T. Chen H. (2017). Tailoring the interfacial chemical interaction for high-efficiency perovskite solar cells. Nano Lett. 17 (1), 269–275. 10.1021/acs.nanolett.6b04015 27936782

